# The inheritance of social status: England, 1600 to 2022

**DOI:** 10.1073/pnas.2300926120

**Published:** 2023-06-26

**Authors:** Gregory Clark

**Affiliations:** ^a^Department of Economics, University of Southern Denmark, Odense DK-5230, Denmark; ^b^Department of Economics, University of California, Davis, CA 95616; ^c^Department of Economic History, London School of Economics, London WC2A 2AE, United Kingdom

**Keywords:** social mobility, genetic inheritance, status persistence, assortative mating

## Abstract

A lineage of 422,374 English people (1600 to 2022) contains correlations in social outcomes among relatives as distant as 4th cousins. These correlations show striking patterns. The first is the strong persistence of social status across family trees. Correlations decline by a factor of only 0.79 across each generation. Even fourth cousins, with a common ancestor only five generations earlier, show significant status correlations. The second remarkable feature is that the decline in correlation with genetic distance in the lineage is unchanged from 1600 to 2022. Vast social changes in England between 1600 and 2022 would have been expected to increase social mobility. Yet people in 2022 remain correlated in outcomes with their lineage relatives in exactly the same way as in preindustrial England. The third surprising feature is that the correlations parallel those of a simple model of additive genetic determination of status, with a genetic correlation in marriage of 0.57.

## Significance

There is widespread belief across the social sciences in the ability of social interventions and social institutions to significantly influence rates of social mobility. In England, 1600 to 2022, we see considerable change in social institutions across time. Half the population was illiterate in 1,800, and not until 1,880 was compulsory primary education introduced. Progressively after this, educational provision and other social supports for poorer families expanded greatly. The paper shows, however, that these interventions did not change in any measurable way the strong familial persistence of social status across generations.

Using a large genealogical database, which details the family connections of 422,374 people with rarer surnames in England for births from 1600 to 2022, the paper examines patterns of inheritance of social status in both preindustrial and contemporary England. Social status is measured by six outcomes: occupational status, higher education status, literacy, dwelling value, company directorships, and the index of multiple deprivation (IMD) for the residence location. Status correlations are calculated for all these outcomes for relatives up to fourth cousins.

These status correlations reveal four things. The first is that status persists strongly across even very distant relatives, across all measures of status. Even fourth cousins, who shared a common ancestor only five generations earlier, typically show statistically significant correlations in status. The second is that the decline in status correlations with each step outward in the lineage is a constant 0.79, for different measures of status, and for different epochs from 1600 to 2022. The vast social changes in England since the Industrial Revolution, including mass public schooling, have not increased, in any way, underlying rates of social mobility.

The third interesting feature of the correlations are that they conform closely to those predicted by Ronald Fisher in 1918, for familial correlations in the presence of strong assortment in mating (1–5). In particular, the correlation in mating in the genetics underlying social outcomes would have to be 0.57 to generate the persistence rate of 0.79. There is ancillary evidence that the phenotypic assortment in marriage in England for underlying social status is around 0.8, and largely unchanged for marriages 1837 to 2022 (6).

Since these are observational data, there is no proof here that additive genetic transmission causes social status. All we can determine is that whatever social processes are producing the observed outcomes have a form of transmission which mimics that of additive genetic effects, in the presence of the important social institution of strong assortative mating. Two recent whole-genome studies for Britain, however, show correlation in marital partners of genetic predictors of educational attainment that are consistent with the 0.57 correlation implied here (7, 8).

Even if in England, 1600 to 2022, social status was mainly determined by genetic inheritance, this does not in itself imply that social interventions cannot change social outcomes. There has been much recent discussion of an alternative causal path through genetic nurture (9–11). Here, the parental genotype creates a social environment for the child which favors better child social outcomes. Genetics is correlated with social outcomes, partly directly, but substantially through the indirect pathway of family environment.

However, in the case where all the important genetic effects are direct, some of the phenomena cited as evidence of genetic nurture can appear as long as spousal similarity at the genetic level is even stronger than implied by their similarity in a given measure of social status. And we will see below that there is indeed evidence of substantial genetic assortment.

Further, the constancy of the patterns of status persistence across the interval 1600 to 2022 does suggest social interventions have surprisingly modest effects. Before 1870, there was little public provision of education, of health care, or of income support. Families largely depended on their own resources. Since 1920, there have been increasing levels of public provision of education, health care, and basic needs. These services should have helped, in particular, poorer families (12). Yet, we see no corresponding increase in rates of social mobility.

## Results

As Fisher demonstrated in 1918, the expected phenotypic correlation between relatives for a trait with an additive genetic architecture is equal to the heritability of the trait, *h*^2^, times a coefficient depending on the genealogical relationship between the relatives and the extent of assortative mating. The details are shown in Table 1. These correlations are derived in the study of Gimelfarb (1981) (4).

**Table 1.** Correlations between relatives with phenotype assortative mating (4)

Relative to child	Correlation	Relative to child	Correlation
Average of parents	$h^{2}$	Ave. grandparents	$h^{2}\left(\frac{1+m}{2}\right)$
Full sibling	$h^{2}\left(\frac{1+m}{2}\right)$	Single parent	$h^{2}\left(\frac{1+r}{2}\right)$
Uncle/aunt	$h^{2}{\left(\frac{1+m}{2}\right)}^{2}$	Single grandparent	$h^{2}\left(\frac{1+m}{2}\right)\left(\frac{1+r}{2}\right)$
Cousin	$h^{2}{\left(\frac{1+m}{2}\right)}^{3}$	Cousin removed	$h^{2}{\left(\frac{1+m}{2}\right)}^{4}$
Second cousin	$h^{2}{\left(\frac{1+m}{2}\right)}^{5}$	Second cousin rem.	$h^{2}{\left(\frac{1+m}{2}\right)}^{6}$
Third cousin	$h^{2}{\left(\frac{1+m}{2}\right)}^{7}$	Third cousin rem.	$h^{2}{\left(\frac{1+m}{2}\right)}^{8}$
Fourth cousin	$h^{2}{\left(\frac{1+m}{2}\right)}^{9}$		

Note: *m* is thecorrelation between the genetic values of the parents, *r* the correlation between the phenotypic values. *h* ^2^ is the heritability of the trait (i.e., the fraction of its variance attributable to genetic causes). These expressions assume that assortative mating occurs with respect to the observed phenotype, not an underlying latent trait or the genetic value itself.

The key parameter determining long-term persistence of familial correlations is *m*, the correlation between the genetic values of spouses. With no assortment, the expected correlation of a trait, even with a heritability of 0.7, for fourth cousins, would be 0.001. Even for second cousins, it would be only 0.02.

Using observed correlations in status across relatives shown in Table 2, *m* and *h^2^* can be estimated. For all but linear descendants of one parent, the expected correlation on the Fisher formulae, $\rho_{n,}$ has the form (from Table 1)

[1] $${ln(\rho }_{n})=\;\ln\left(h^{2}\right)+\;nln{\left(\frac{1+m}{2}\right)}^{}.$$

**Table 2.** Social status correlations by familial connection, England, 1600 to 2022

Outcome	ModStat	House value	IMD	CoDir	OccStat	OccStat	HighEd	HighEd	Literacy
Birth period	1910–1997	1910–1997	1910–1997	1910–1997	1780–1859	1860–1919	1780–1859	1860–1919	1725–1869
Pairs observed	117,489	117,625	118,182	237,933	140,616	366,969	134,664	368,030	55,067
Correlations									
Full sibling	0.375	0.336	0.269	0.168	0.578	0.522	0.479	0.326	0.431
Child	0.396	0.360	0.330	0.132	0.595	0.512	0.538	0.374	0.336
Sibling-rem	0.256	0.246	0.172	0.057	0.502	0.380	0.382	0.228	0.273
Grandchild	0.313	0.278	0.245	0.083	0.451	0.362	0.381	0.249	0.196
Cousin	0.208	0.211	0.144	0.061	0.431	0.299	0.291	0.173	0.234
Cousin-rem	0.133	0.147	0.098	0.016	0.341	0.274	0.234	0.162	0.191
Cousin2	0.133	0.141	0.073	0.069	0.266	0.234	0.176	0.186	0.200
Cousin2-rem	0.084	0.084	0.054	0.028	0.189	0.203	0.032	0.125	0.222
Cousin3	0.097	0.100	0.052	0.053	0.139	0.174	0.071	0.085	0.150
Cousin3-rem	0.059	0.069	0.013	0.016	0.070	0.111	0.094	0.032	0.052
Cousin4	0.074	0.078	0.028	0.021	0.071	0.066	0.080	0.014	0.107
Unrelated	−0.002	−0.004	0.000	0.001	−0.002	−0.002	0.005	−0.004	0.001

Notes: “-rem” indicates “once removed.” Cousin 2, 3, and 4 indicate second, third, and fourth cousins, respectively. ModStat is a PCA index that combines House Value, IMD, and the Company Director indicator (CoDir). “OccStat” is occupational status, “HighEd” is an indicator for higher education. “Pairs Observed” is the total number of pairs of relatives used in estimating the parameters of Eq. **3**. *SI Appendix*, Table S1 shows the number of observations for each individual pairing.

In the other cases, the expected correlation is

[2] $${ln(\rho }_{n})=\;\ln\left(h^{2}\right)+\;nln\left(\frac{1+m}{2}\right)+\;ln\left(\frac{1+r}{1+m}\right),$$

where r is the phenotype correlation between spouses.

This means that we can estimate *m* and *h^2^* from the parameters of a linear regression

[3] $${ln(\rho }_{n})=a\;+\;\ln\left(b\right)n\;+cd_{\mathrm{lin}},$$

where $d_{\mathrm{lin}}$ is an indicator which is one for the cases where the phenotype marital correlation appears. $b=\frac{1+m}{2}$ is the persistence rate of the correlation as we move one step down the family tree, or one step across between full siblings. To allow for the much greater SE of the estimated ${ln(\rho }_{n})$ for more distant relatives, expression (3) was fitted using weighted least squares, weighted by the inverse squared SE of each correlation.

Once *m* is estimated, we can graph the link between the implied fraction of shared genotype and the correlation of social outcomes.

**Fig. 1.**
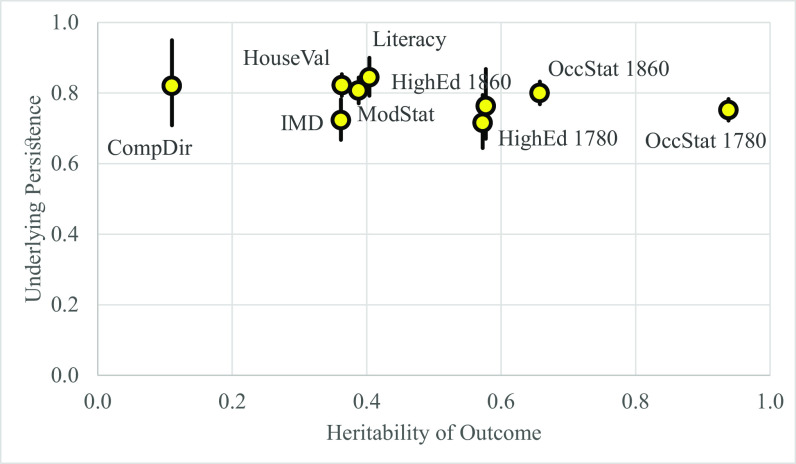
Estimates of persistence versus heritability, births 1780 to 1997. Notes: Company Director (2002 to 2022), “CompDir;” Ln House Value, 1999 to 2022, “HouseVal;” Index of Multiple Deprivation, 1999 to 2022, “IMD;” Modern Social Status, 1999 to 2022, “ModStat;” Occupational Status births, 1780 to 1859, “OccStat1780;” Occupational Status births, 1860 to 1919, “OccStat1860;” Higher Education births, 1780 to 1859, “HighEd1780;” Higher Education births, 1860-1919, “HighEd1860;” Literacy Marriages, 1754-1879, “Literacy.” Lines indicate 95% CIs for the estimates.

Fig. 1 shows the estimated values of $b=\left(\frac{1+m}{2}\right)$ for each of the outcome measures from Eq. **3**. It also shows the 95% CI of each estimate. This is plotted against the estimated heritability of each trait, also from ref. 3. *SI Appendix*, Table S2 gives the estimated values of *b, m,* and *h^2^* from these estimates, as well as the R^2^ of the fit, which averages 0.88.

As Fig. 1 shows, the estimates of *b*, social status persistence, cluster around 0.785 for all the nine measures, even though the measured heritability of the measured traits varies substantially. The high R^2^ of the fit implies that the Fisher formulas predict well the correlations. Fig. 1, which shows the same underlying rate of social mobility from the eighteenth century to the present, suggests that possibly all social statuses show the same persistence parameter of 0.79. If this is through additive genetic transmission, then also throughout this period, marital partners had to be correlated 0.57 on the relevant genetics.

If marital assortment is on an observed phenotype, then

[4] $$m=rh^{2},$$

Thus, with genetic transmission, the marital correlation on the phenotype couples are matching on, *r*, has to be even larger than the 0.57 implied for the genotype. However, as we shall see below, the various measures of social status in Table 2 are just imperfect partial measures of some general social ability that couples sort on. For these partial measures of the sorting phenotype, it will not hold that $m=rh^{2}$ . Both *r* and $h^{2}$ will be attenuated for these imperfect phenotype measures. Thus for literacy, the observed marital correlation in the lineage is only 0.41, less than the implied genotype correlation in social aptitude of 0.57.

In Table 2, we estimate the genetic link between individuals by their closest genealogical connection. However, if siblings sometimes marry siblings, and cousins sometimes marry each other, then some individuals labeled as first, second, etc., cousins will be more closely connected genetically than the standard cousin. The question in practice is how significantly in England did the marriage of relatives increases the shared genetics of individuals in a family tree above those implied by Table 1?

The biggest contributor to a greater genetic connection than predicted by looking just at the closest common ancestor will be the frequency of cases where siblings marry siblings (this cannot be that uncommon since my own maternal grandparents had siblings who married each other). In this case, for example, the shared genes of cousins (who are now double cousins) will be ${\left(\frac{1+m}{2}\right)}^{3}/\left(1-{\left(\frac{1+m}{2}\right)}^{3}m^{2}\right)$ instead of ${\left(\frac{1+m}{2}\right)}^{3}$ (4). Based on the estimates of the paper of *m *= 0.57, this implies a shared genotype of 0.484 for single cousins and 0.605 for double cousins.

From the Families of England database, we can estimate the share of all pairs of siblings who married siblings by use of the birth surname of their marriage partners (which will be the same for siblings). A set of 123,737 such sibling pairs show 1,773 with marital partners with the same surname. A random assignment of these surnames to each other produces only 97 matches. The implication is that as many as 1,676 (1.35%) of the sibling pairs married partners who were also siblings.

However, based on *m *= 0.57, this would imply that the estimate of persistence of 0.79 from varieties of cousins would be inflated above the true persistence rate by only 1.0034 as a result of such extra genetic connections. This is not a significant inflation of persistence.

The major second source of inflated genetic correlations will be cousin marriage. The frequency of this can also be estimated from the surnames of marital partners. In 25% of cousin marriages, the parties to the marriage will share a surname (when their fathers are brothers). Thus, we can estimate the proportion of cousin marriages from the excess fraction of marriages where spouses share a surname compared with the expected share based on the relative surname frequency among marital partners. For marriages in England, 1837 to 2021, on average, there were 0.35% more marriages between same surname partners than would be created by random matching between parties. This in turn implies that 1.42% of marriages were between cousins.

For second cousins who are the product of cousin marriages, the shared genotype will be again inflated above the single second cousin share of ${\left(\frac{1+m}{2}\right)}^{5}$ , but the precise factor has not been derived in the literature. However, if we assume that the inflation in the genetic share was similar to the 1.25 in the first cousin case above, then the observed frequency of cousin marriage implies that the actual shared genotype will be inflated by a factor of only around 1.0036 for second cousins and beyond.

The combination of both these sources of additional shared components in the genotype would inflate the shared genotype above that calculated from Table 1 by 0.34% for cousins, and around 0.7% for second cousins and beyond. This will not significantly inflate the estimates of *m* in the paper.

**Fig. 2.**
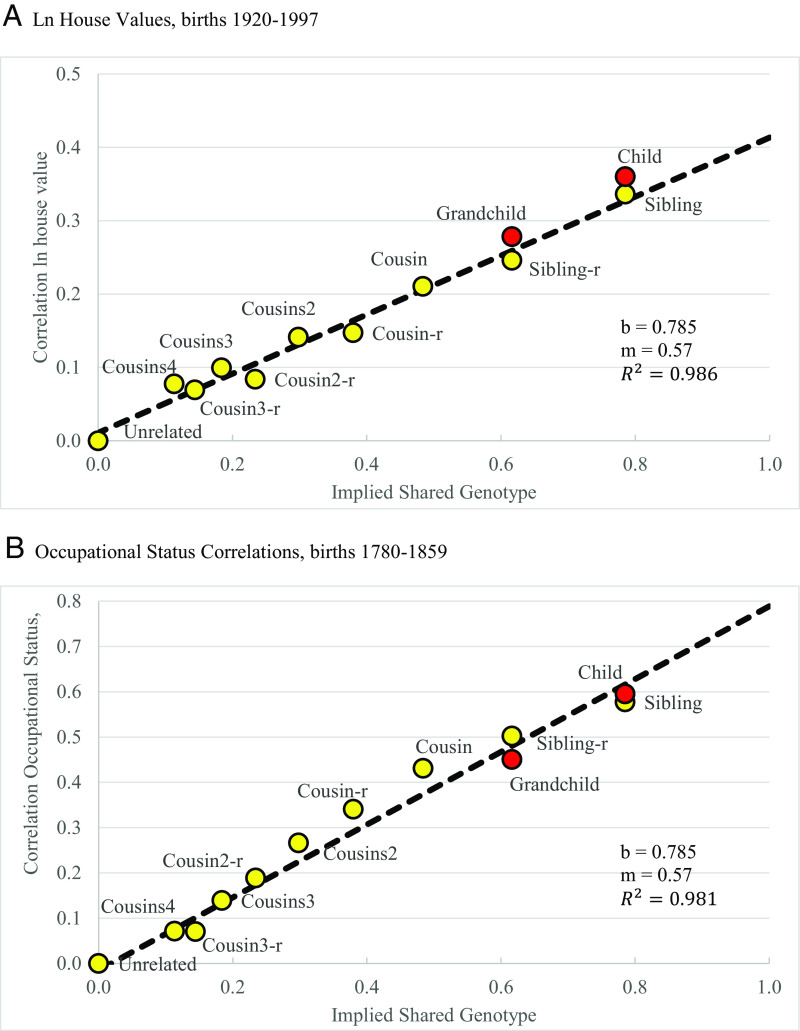
Social status correlations and implied shared genotype. Notes: (*A*) shows correlations of ln house values 1999 to 2022, for births 1920 to 1997, for different relations, and the estimated shared genotype on the assumption that m = 0.57. (*B*) shows occupational status correlations for births 1780 to 1859. The dashed line in each case shows the OLS fit to these data. The R^2^ reported is for this fitted line. The child and grandchild correlations will potentially deviate from the fitted line.

Fig. 2 illustrates how well the assumption of additive genetic determination of social status, with marital assortment at 0.57, describes the data for two example outcomes. With the assumption that *m* = 0.57, we can arrange the various pairs of relatives in terms of their shared genotype on the horizontal axes. Then, on the vertical axis, we can plot the relevant correlation. In Fig. 2*A*, this is the log house value 2017. As noted above, the house value is normalized by region to remove regional effects. House value is serving here as an indicator or the income of the family. The fit is based on 117,625 house value correlations between different sets of relatives.

As would be predicted with additive genetic transmission of outcomes, there is a clear linear relationship in Fig. 2*A*, between the implied genotype share of relatives and the house value correlation. The R^2^ of the fitted line here is 0.986, and the intercept with the vertical axis is not statistically different from 0.

This linearity of the relationship in Fig. 2 further emphasizes the stability of the persistence rate b over generations. If persistence from one generation to the next was much higher in earlier years, then for fourth cousins, where on average in these data the common ancestor was born in 1804, the measured correlation now would be above the fitted line in the figure for more distant relatives. Note also that even for fourth cousins in 1999 to 2022, who would likely have no social interaction, the correlation in house values within regions is both quantitatively and statistically significant.

Fig. 2*B*, similarly shows the close correlation between the implied fraction of shared genes (assuming *m* = 0.57) and the correlation of occupational status for men born 1780 to 1859. The fit here is based on 140,616 pairs of occupational status. Again the relationship is linear, as additive genetic transmission would imply. Again, the OLS-fitted line intercepts the vertical axis close to 0. And again this implies a stability in the persistence of status across generations all the way from 1,678 or earlier, when on average fourth cousins had a common ancestor, to 1,859.

In the Supporting Information are shown the correlations of status and implied shared genotype for the other seven status measures, assuming genetic marital assortment of 0.57 (*SI Appendix*, Figs. S1–S7). The R^2^ of the fit varies with the numbers of observations, and the heritability of the trait. But it averages 0.95. *SI Appendix*, Figs. S1–S7 look similar to the ones presented here, and show the same consistent pattern in inheritance.

We can also test whether any of the relationship correlations, such as between cousins, systematically deviate from the predicted additive genetic pattern of Table 1. This is done for the nine relatedness types shown in Table 3. The test was implemented by estimating the basic weighted least squares regression (3) on the log of correlation versus degree of relatedness for all the nine social outcomes, but adding in each regression an indicator for one of the nine specific relationships (sibling, cousin, etc.). This produced 81 estimated indicator coefficients and associated SE. For each relationship pair, there was estimated the following regression:

**Table 3.** Deviations of relationship types from Fisher regression line

Relation	Average	SE	t-statistic
Sibling	0.044	0.055	0.81
Sibling-rem	−0.068	0.036	−1.86
Cousin	0.002	0.024	0.06
Cousin-rem	−0.069	0.040	−1.74
Cousin2	0.083	0.055	1.51
Cousin2-rem	−0.097	0.096	−1.01
Cousin3	0.181	0.097	1.86
Cousin3-rem	0.011	0.090	0.13
Cousin4	0.210	0.132	1.59

Notes: “-rem” indicates “once removed.”

Indicator value = constant (weighted by inverse indicator SE squared)

The estimated values of each constant are shown in Table 3. The coefficients reported are the fractional deviation of each relationship from the regression line. For no single relationship correlation was there evidence of significant systematic deviation from the correlation predicted by genetic relatedness across the nine social outcomes.

However, the average coefficient for a relative of the same generation was 10% higher than the regression prediction, and for a relative one generation apart, 6% lower than the regression prediction. These collective differences are statistically significant. Note, however, that since average correlations of relatives varied by age and observation period, this effect may just reflect closer age and time period concordance of relatives who are from the same generation.

The lineage database also contains a large number of observations on wealth at death for men and women dying 1800 to 2022. This measure was not included in Fig. 1 and Table 2 because it clearly involves the nongenetic transfer of wealth between generations. For richer families, that transfer was also affected by social elements such as the number of children in a family, or by the gender of the child. Wealth inheritance also shows a significant asymmetry between men and women in a way that is inconsistent with additive genetic transmission. The implied persistence of wealth by generation (b), however, is even higher than for the measures used here, being 0.84 for births 1780 to 1859, and 0.86 for 1860 to 1919.

The finding of an intergenerational persistence rate of 0.79 that is stable over time is buttressed by surname status studies carried out by the author and collaborators. Rarer surnames often deviate in average social status from the social mean. Surname inheritance in a society such as England follows the same pattern as the y chromosome. Thus, the rate of movement of surname status toward the social average should show a persistence across each generation of about 0.79. For England, there is exactly such a surname status persistence, an unchanged persistence from the seventeenth century until now (13, 14).

## Data

Table 2 summarizes the correlations of social status outcomes for nine measures of social status. The number of pairs of outcomes from which these correlations are derived is given in *SI Appendix*, Table S1. For the current period, births 1910 to 1997, there are, for both genders, estimated log house value, normed to 2017, the IMD, Company Director, and a combined social status score from these first three measures. For these contemporary correlations, all the data are included, but the common ancestor between two individuals must be born 1780 or later. The elite lineages cannot be used for ancestors born before 1780, since they were selected on the basis of the status of ancestors born in the period 1780 to 1840.

For men born 1860 to 1919 and 1780 to 1859, we have both occupational status and attainment of higher education. Women in England were not admitted to most universities and professional qualifications until 1920 or later, so though there were highly educated women, there is no formal record of that. Middle and upper class women typically did not work outside the home, so occupational status measures for women before 1920 are not very useful indicators of social status. Finally, for men and women born 1725 to 1869, we have literacy measured at marriage. This was recorded for all marriages 1754 and later.

Table 2 also shows that in all cases where individuals were randomly assigned partners from another surname lineage, and so unrelated, the estimated correlation was close to 0. There is nothing in the structure of the data that is spuriously creating correlations even between unrelated individuals.

### Additional Tests.

Another implication of direct additive genetic transmission is symmetry of mothers and fathers in transmitting status to children. As noted above, for much of this period 1600 to 2022, we do not observe social status outcomes (except literacy) for women. But, we can proxy the implied status of mothers and fathers by using the status of the maternal and paternal grandfathers. We can then estimate the parameters $b_{f}$ and $b_{m}$ in the following equation:

[5] $$y_{c}\;=a+b_{f}y_{\mathrm{g f}}\;+\;b_{w}y_{\mathrm{gm}}\;+e,$$

where $y_{c}$ is the social outcome for the grandson, $y_{\mathrm{gf}}$ is the outcome for the paternal grandfather, and $y_{\mathrm{gm}}$ is the outcome for the maternal grandfather. For births in the period 1780 to 1919, we have as grandfather outcomes occupational status, higher education, and wealth at death. In addition for 1754 to 1889, we have mother and father literacy at marriage, and child literacy at the child’s marriage, which allows a direct estimate of the relative predictive ability of mother versus father literacy for both daughters and sons.

**Fig. 3.**
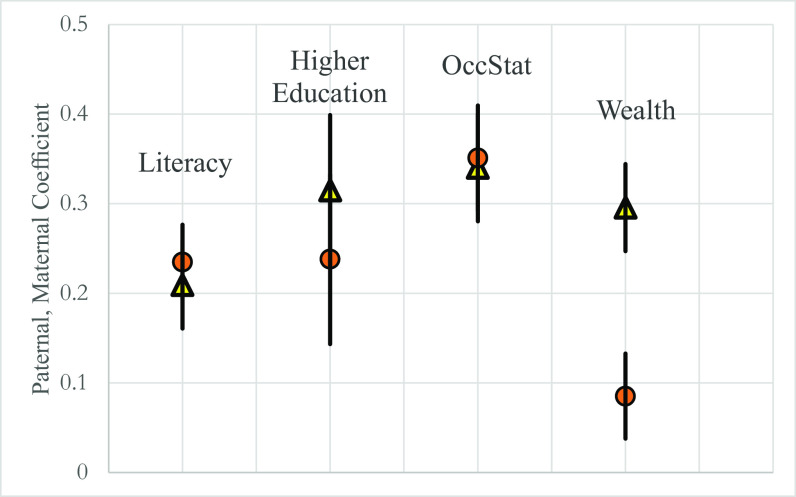
The comparative influence of mothers and fathers. Notes: Ο – mothers, Δ – fathers. 95% CIs are indicated by bars. CIs generated clustering on fathers. For higher education, occupational status, and wealth, the status of father and mother is measured by their fathers’ status.

Fig. 3 shows the estimated coefficients from ref. 5, and directly for literacy. For literacy, higher education attainment, and occupational status, there is no significant difference in the predictive effect of father versus mother status (or that of their fathers). But the wealth of the paternal grandfather is three times as large as that of the maternal grandfather in predicting child wealth.

Fig. 3 also shows that the focus of the One-Name Studies lineages on the patriline will not exaggerate estimates of status persistence across generations, except in the case of wealth. Persistence is just as strong in the matriline as in the patriline.

If all status transmissions between fathers and sons were direct transmissions through genetics, then the phenotype correlation between sons and fathers should be the same for those whose fathers died early in their childhood as for those whose fathers were alive when they reached age 21. If, however, indirect transmission through the effects of parent genetics on child environment is a major source of status transmission, then by implication, children with dead fathers should correlate less closely with their biological fathers. They will be raised by their mothers, or their mothers and stepfathers. But mothers only correlate in underlying phenotype with fathers by a factor r, and stepfathers by an even smaller factor of r^2^.

**Fig. 4.**
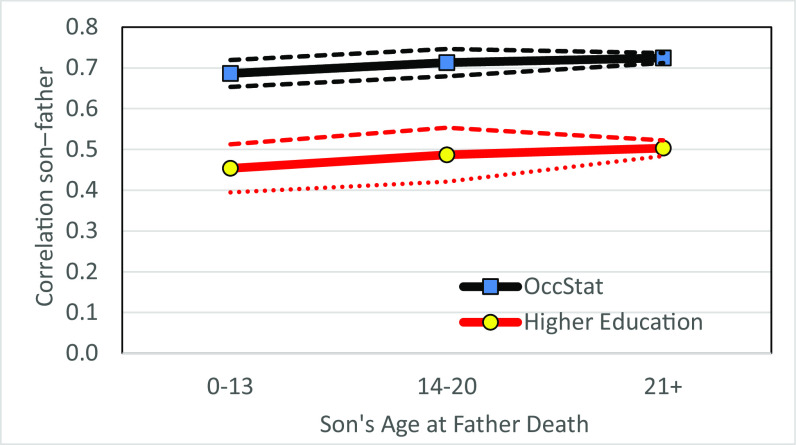
Correlation with biological father and age at father’s death. Notes: Sons born from 1780 to 1919. Correlation of occupational status and higher education. Dashed lines indicate 95% CIs. Families of England database.

In the FOE database, 10% of sons born 1780 to 1919 had fathers who died when they were aged 0 to 13, and a further 8% had fathers who died when they were aged 14 to 20. For these sons, we observe adult occupational status and higher education. For these two outcomes, there is an indication that correlations of sons with dead fathers are slightly lower than those of sons with living fathers. But the effect is so modest that it is not statistically significant at even the 5% level. Fig. 4 shows these estimates and their CIs. This is consistent with the direct genetic pathway being the predominant determinant of social status.

### Spousal Correlation in Marriage.

Until recently, the finding that an additive genetic model of status determination, combined with the social parameter of a spousal correlation in the underlying genetics of 0.57, would have been dismissed on the grounds that spousal correlations in genetic values could not be so high. A typical phenotype where spousal correlations have been measured is years of education, and here the correlations for the modern United Kingdom are typically 0.4 to 0.5 (7, 8, 15, 16). On the normal assumption that $m=\;h^{2}r$ , this implies a correlation in the relevant genes of less than 0.25 (7, 8, 15, 16).

However, two recent studies of the genetic predictors of educational attainment (measured as years of schooling) both imply that the spousal correlation in the genetics relevant to educational attainment is much higher than 0.25. In the first study, based on 7,780 UK Biobank couples with measures of educational attainment, the spousal phenotype correlation was only 0.41 (s. e. 0.011). However, the correlation across the same couples at trait-associated loci for educational attainment was significantly higher, 0.654 (s. e. 0.014) (7).

The second study showed a phenotype correlation in years of education of 0.43 (s. e. 0.017) within 2,465 couples from the United Kingdom. There was, however, an unexpectedly high (0.175) correlation (s. e. 0.020) in the polygenic index for educational attainment (8). Since the polygenic index is a noisy measure of the full genetic educational potential, the full correlation will be significantly higher than this measured correlation.

If we take the analogous case of height, also reported in this paper, the phenotype correlation between spouses was 0.290 (s.e. 0.018), but the polygenic index index correlation was only 0.106 (s.e. 0.020) (8). Since height has a heritability of 0.8 and is largely genetically determined in high-income societies, the true genetic correlation between partners in height would thus be 0.236. This implies that the polygenic index correlation for height between partners has to be multiplied by 1.65 to 3.27 to estimate the full genetic correlation. If we apply this same adjustment to the measured genetic correlation between spouses for educational attainment, then the implied actual correlation averages 0.39, with a 95% CI of 0.29 to 0.57. The height polygenic index is based on larger samples, and height as a phenotype has less noise than educational attainment. So, the 0.175 genetic correlation observed between partners for educational attainment is potentially consistent with a true genetic correlation of 0.57 (17).

Another recent study for Norway, with 26,681 pairs of partners and 2,170 pairs of siblings, found a 0.42 phenotype correlation between partners in years of education, but an estimated 0.37 genetic correlation for educational attainment between partners. This is lower than the UK estimates, but the 95% CI for this estimate was 0.21 to 0.67. In line with this partner correlation, the sibling genetic correlation was estimated as 0.68 (95% CI: 0.61 to 0.75). Comparison of the genetic similarities of partners and siblings implied that assortative mating at the observed level had taken place for at least five generations in Norway (16).

Thus the evidence, at least for the modern United Kingdom, is that parents are matching much more strongly on a latent social ability phenotype than they are on the observed phenotypes such as years of education, occupational status, or income. This strong matching then makes possible the high observed genotype correlation.

We can find evidence in marital records for England and Wales from 1837 to 2022 for just such strong latent status phenotype matching (5). As noted above, these marital records, collected again by amateur genealogists, show occupations for grooms and brides and their respective fathers. Suppose that grooms and brides match in marriage to some social status phenotype they observe, with a correlation, r. Suppose also we only have noisy measures of this phenotype, such as years of education, or an occupational status index. In that case, the observed phenotype correlation in marriage will be biased downward by some factor θ<1 . But suppose also that both bride and groom correlate in their true social phenotype with a correlation of β with their respective fathers. This implies that the observed correlation of groom to his father will be θβ . The observed correlation of the groom to their father-in-law, if the matching in marriage is just bride to groom, will be θrβ . This implies that the true correlation between bride and groom in their social phenotype can be calculated as

[6] $$\frac{groom-father-in-law\;correlation}{groom-father\;correlation}\;=\;\frac{\theta r\beta }{\theta \beta }\;=\;r.$$

For marriages in England and Wales, from 1837 to 2022, this underlying correlation is consistently close to 0.79 across all periods, as is shown in Table 4 (6). It may be objected that if the groom or the groom’s father is also matching directly to the father-in-law, the measured marital correlation will be driven upward. However, this estimation produces the same marital correlation in cases where the father-in-law is dead at the time of the marriage, or in cases where the father is dead. In such cases, we would expect less groom–father-in-law matching if such matching was occurring, and consequently a lower estimated marital correlation. We observe no sign of that.

**Table 4.** Implied underlying phenotype correlation in marriage, 1837 to 2021

Period	N	Status index	$\rho_{gf}$	$\rho_{gfinl}$	r
1837–1859	343,623	HISCAM	0.631 (0.001)	0.480 (0.002)	0.771 (0.004)
1860–1899	438,725	HISCAM	0.601 (0.001)	0.464 (0.001)	0.772 (0.004)
1900–1940	174,474	HISCAM	0.498 (0.002)	0.384 (0.002)	0.771 (0.004)
1940–1979	47,033	CAMSIS	0.424 (0.004)	0.346 (0.004)	0.816 (0.017)
1980–2021	10,444	CAMSIS	0.339 (0.009)	0.275 (0.009)	0.812 (0.045)

Notes: *gf* = groom–father, *gfinl* = groom–father-in-law. SE in parentheses.

Source: Clark and Cummins, 2022, Tables 3 and 4 (6).

A marital correlation in a latent social status phenotype of 0.79 is compatible with a correlation in social status genetics of 0.57. It would rely on a heritability of the underlying social status of 0.72, which is high but similar to that for height. Thus, the evidence on strong latent phenotype matching in marriage throughout the years 1837 to 2022 is consistent with the evidence above of strong and stable genetic matching throughout this period. Collado, Ortuño-Ortín, and Stuhler, 2022, find similar evidence of strong marital assortment in latent social abilities for recent generations in Sweden (18).

## Materials and Methods

The lineage connections in the database were largely identified by amateur genealogists constructing family trees, using publicly available birth and baptism, marriage, death, census, and probate records. Family lineage studies can involve significant problems of selectivity, where more notable ancestors, or those leaving descendants, are more often included. To avoid such problems of selectivity in who gets included in a family tree, the lineages used here are mainly those constructed by the members of the Guild of One-Name Studies (19). Guild members aim to include all persons with a chosen rare surname––Argall, Errey, Byatt, etc.—in their lineages. This avoids the problem of selective inclusion, though because surnames are preserved at marriage only for males, it does focus on the patriline. However, as was shown above for most outcomes except wealth, intergenerational transmission of status is symmetric on the matriline and the patriline. Also comparison of wealth, literacy, and occupational status for the lineages used here, detailed in supporting information, suggests that these lineages are only of modestly higher than average status across the years 1800 to 2022.

To many of these lineages derived from Guild members have been added additional information on social outcomes derived from census records 1841 to 1911; from the 1939 population register; marriage records 1837 to 2022; ship passenger records; the electoral rolls 1999 to 2022; registers of company officers; matriculation records for Oxford, Cambridge, Durham, and London Universities; the medical register 1857 to 2022; armed forces appointments; and members of engineering societies. Again, all these are publicly available sources.

For people in the most recent years, the electoral rolls 1999 to 2022 reveal the address of many individuals (20). This makes it possible to estimate the value of the house people were living in, by postcode, using the UK Land Registry data on sales 1995 to 2017 (where the typical postcode covers 40 houses) (21). Since the data show that people show strong geographic persistence, and since house values vary substantially by region in England and Wales, we normalize house values in the sample to their deviation from the average house value across six regions (North, Midlands, Wales, East and South East, London, and South West). From the address, we also observe the social status of the local area (around 1,000 households) as expressed by the Index of Multiple Derivation (IMD) for 2019 (22). Independently, we can identify if a person alive 2002 and later was a Company Director, from the Director’s Register (23). To get an independent measure of status from the address, we included only individuals aged 24 and above who were not at the same address as a parent. Using the three measures––house value, IMD, and Company Director—we derive using principal component analysis a more general measure of social status, “Modstat,” for those living 2002 and later.

In earlier years, we have two measures of status which apply only to males. The first is occupational status. An index of social status by occupation was estimated from 1.4 million marriage records 1837 to 1939 which give occupations at marriage for the groom, his father, and his father-in-law (24). Status was assigned to occupations in such a way as to maximize the father–groom and father-in-law–groom correlations. The second is whether a person had attained higher education such as attending university or a military academy, and/or qualifying as an accountant, attorney, doctor, engineer, or clergyman. We have a further measure, literacy at marriage, which applies to both men and women marrying 1754 to 1889. This is inferred from the ability to sign the marriage register. The higher education measure will tend to be informative of educational status for those of higher status, while the signature measure will be informative for those of lower status.

## Supplementary Material

Appendix 01 (PDF)

Appendix 02 (PDF)

Dataset S01 (XLSX)

Dataset S02 (XLSX)

Dataset S03 (XLSX)

Dataset S04 (XLSX)

## Data Availability

The data used in this study, such as modern house values, were all obtained from public records. But since the convention in genealogy is to keep living persons’ information anonymous, anonymized data underlying all estimates in the paper are available in the Supporting Information.
