# Supplementary material for: The inheritance of social status: England, 1600 to 2022
Source: Proc Natl Acad Sci U S A. 2023 Jun 26;120(27):e2300926120. doi: 10.1073/pnas.2300926120 (PMC10319028; doi:10.1073/pnas.2300926120)
Supplement: Supplementary file 1 — Appendix 01 (PDF) [file pnas.2300926120.sapp1.pdf]

The Inheritance of Social Status: England, 1600-2022, Supporting Information

Gregory Clark

1. Estimations of the Fisher relationships

2. Marital Assortment

3. The Families of England database

4. Replication Files

1. Estimations of the Fisher relationships

Table S1: Social Status Pairs by Familial Connection, England, 1600-2022

**Outcome**	**Modstat**	**House value**	**IMD**	**CoDir**	**Occstat**	**Occstat**	**HighEd**	**HighEd**	**Literacy**
Birth Period	1910-97	1910-1997	1910-1997	1910-1997	1780-1859	1860-1919	1780-1859	1860-1919	1725-1869
**Pairs Observed**									
Full Sibling	7,840	7,840	7,895	17,856	10,585	15,063	10,772	15,806	5,856
Child	10,678	10,678	10,678	17,774	8,530	15,695	8,337	15,394	7,467
Sibling-rem	9,101	9,101	9,160	20,573	12,300	35,421	12,030	34,874	7,081
Grandchild	2,285	2,285	2,314	4,801	4,734	16,031	4,619	15,661	4,450
Cousin	8,523	8,523	8,523	18,475	14,941	27,022	14,635	26,829	4,831
Cousin-rem	10,843	10,904	10,904	25,887	12,744	52,557	12,319	51,001	6,215
Cousin2	10,083	10,087	10,165	21,225	16,531	35,135	15,935	33,813	3,289
Cousin2-rem	15,708	15,708	15,856	33,422	12,843	59,134	12,212	57,201	3,645
Cousin3	15,570	15,641	15,761	26,657	16,493	35,626	15,349	33,900	1,778
Cousin3-rem	17,843	17,843	17,911	37,889	13,533	57,299	12,503	53,447	2,830
Cousin4	9,015	9,015	9,015	13,374	17,382	33,049	15,953	30,104	1,600

Notes: "-rem" indicates "once removed". Cousin2, 3 and 4 indicates second, third and fourth cousins. Modstat is a PCA index that combines House Value, IMD and the Company Director indicator (CoDir). "Occstat" is occupational status, "HighEd" an indicator for higher education. "Pairs Observed" is the total number of pairs of relatives used in estimating the parameters of equation (3).

Table S2: Summary of Estimates of *b*, *m*, and h^2

Outcome	Birth Period	Pairs	Gender	b	m	h^2	R^2
Modern Status	1910-97	117,489	both	0.769	0.538	0.466	0.973
Normed L(house value)	1910-97	117,625	both	0.798	0.596	0.412	0.978
Index Multiple Deprivation	1910-97	118,182	both	0.732	0.463	0.358	0.989
Company Director	1910-97	237,933	both	0.783	0.567	0.191	0.718
Literacy	1725-1869	48,859	both	0.847	0.694	0.427	0.625
Occupational Status	1780-1859	140,616	male	0.812	0.624	0.722	0.964
Occupational Status	1860-1919	382,032	male	0.786	0.572	0.652	0.931
Higher Education	1780-1859	134,664	male	0.774	0.548	0.627	0.983
Higher Education	1860-1919	368,030	male	0.766	0.531	0.420	0.886
Average				0.785	0.570		0.884

Table S2 reports the details of the estimates of *b, m*, and h^2 from equation (3) for the 9 different outcomes that underly Figure 1, based on the correlations reported in table 2.

Figures S1-S7 show the correspondence between genetic closeness and outcome correlation under the assumption that $m = 0.57$. The horizontal axes show the estimated shared genotype. The vertical axes show the measured outcome correlations. The dashed line shows the OLS fit to this data. In only one case, where the index of multiple deprivation (IMD) is the outcome, does the OLS line fitted to the data show a significant deviation from 0 at the vertical axis.

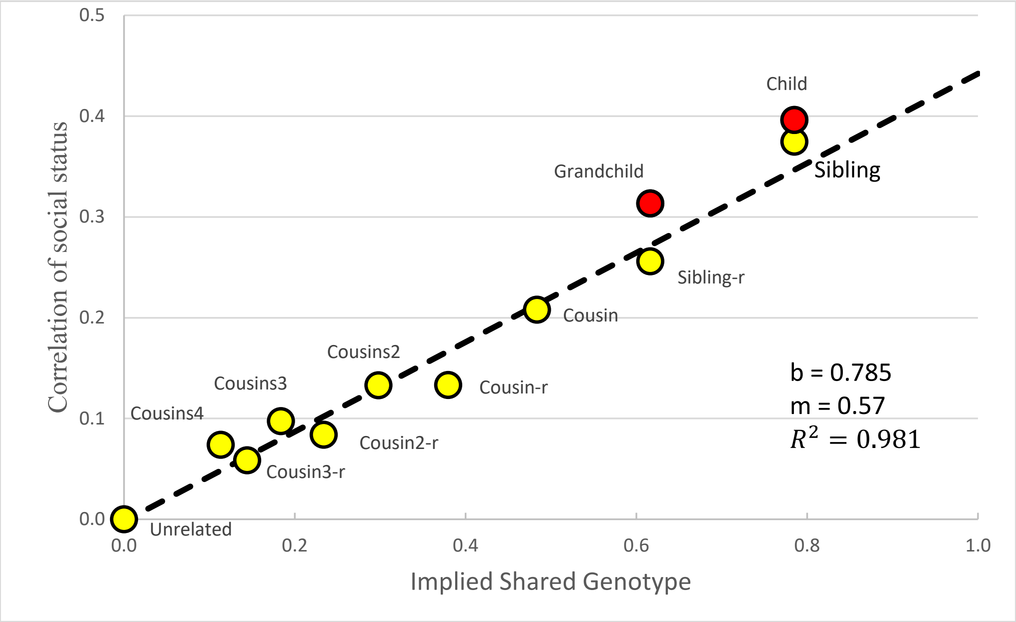

Figure S1. Modstat Correlations and Implied Shared Genotype

Notes: The dashed line shows the OLS fit to this data. The R^2 reported is for this fitted line. The child and grandchild correlations potentially deviate from this fitted line.

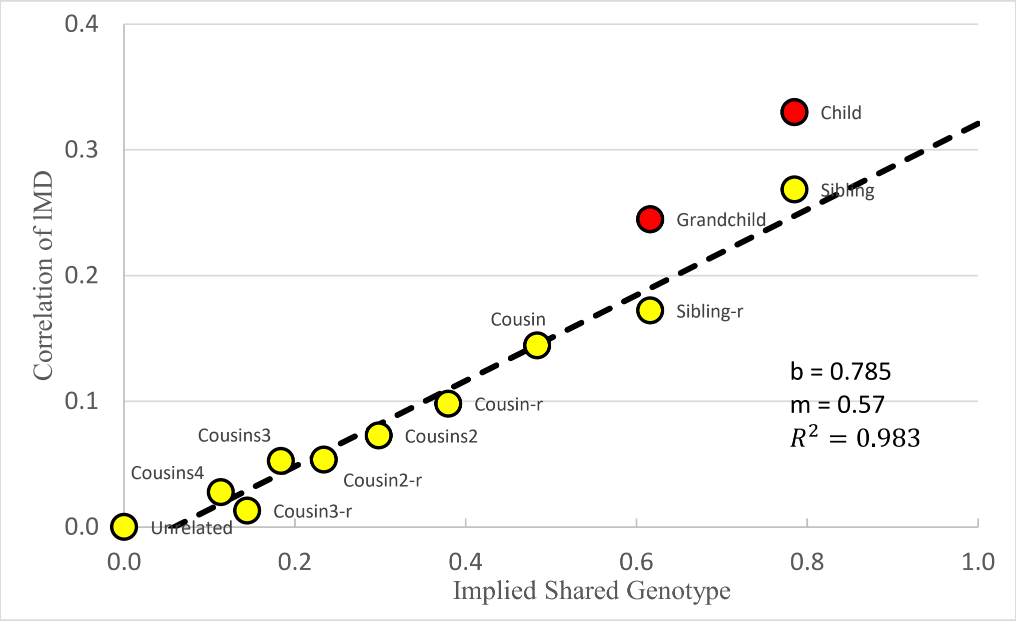

Figure S2. IMD Correlations and Implied Shared Genotype

Notes: The dashed line shows the OLS fit to this data. The R^2 reported is for this fitted line. The child and grandchild correlations potentially deviate from this fitted line.

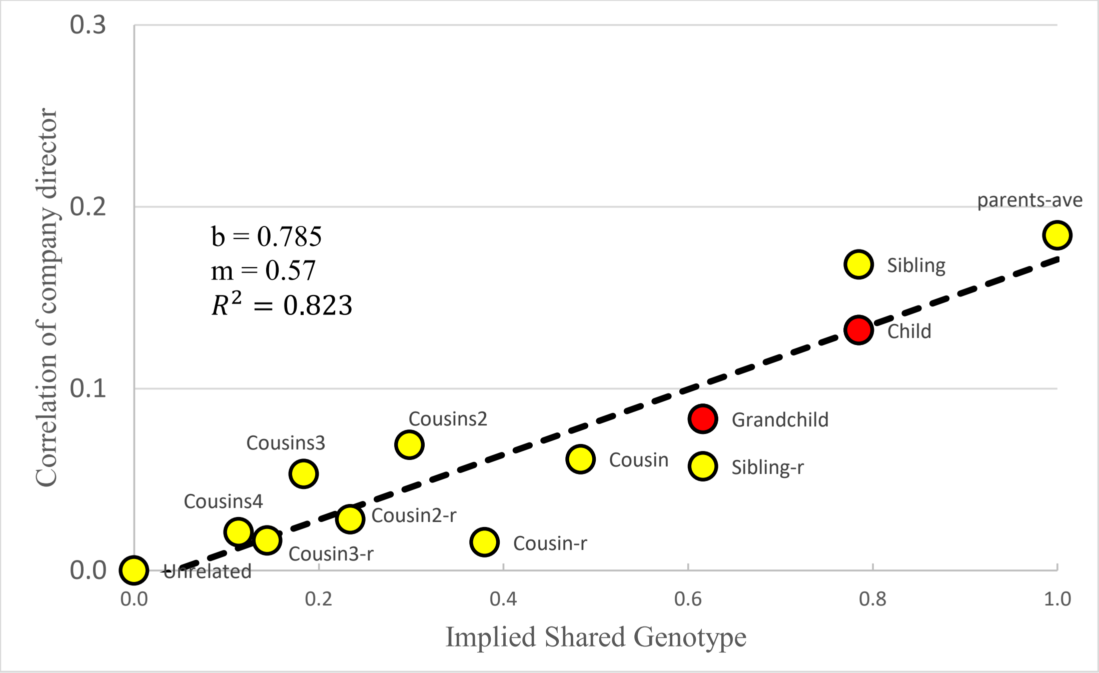

Figure S3: Company Director Correlations and Implied Shared Genotype
Notes: The dashed line shows the OLS fit to this data. The R^2 reported is for this fitted line. The child and grandchild correlations potentially deviate from this fitted line.

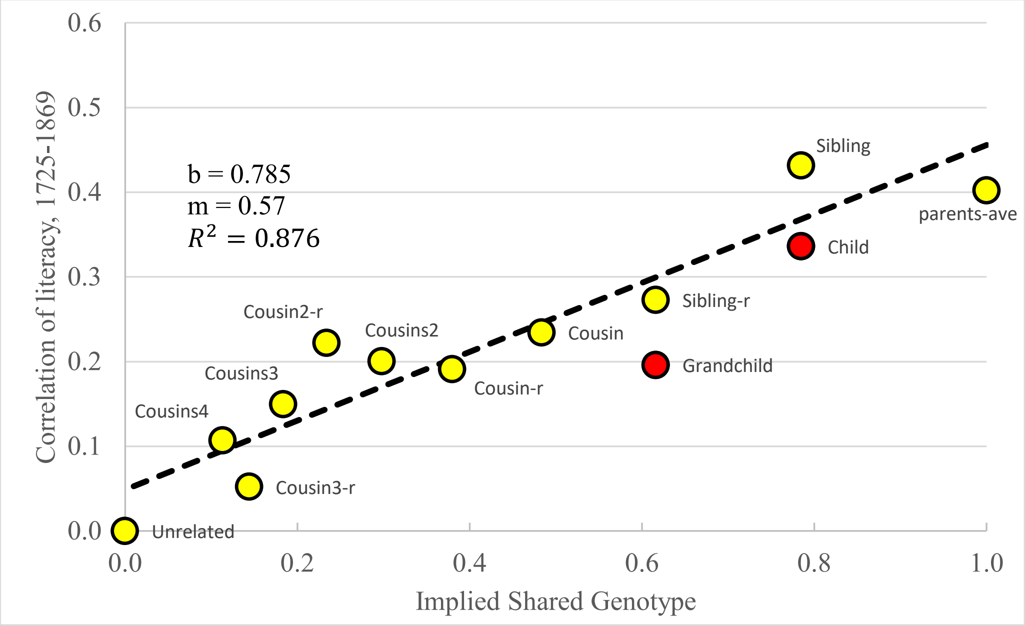

Figure S4: Literacy, births 1725-1862, and Implied Shared Genotype
Notes: The dashed line shows the OLS fit to this data. The R^2 reported is for this fitted line. The child and grandchild correlations potentially deviate from this fitted line.

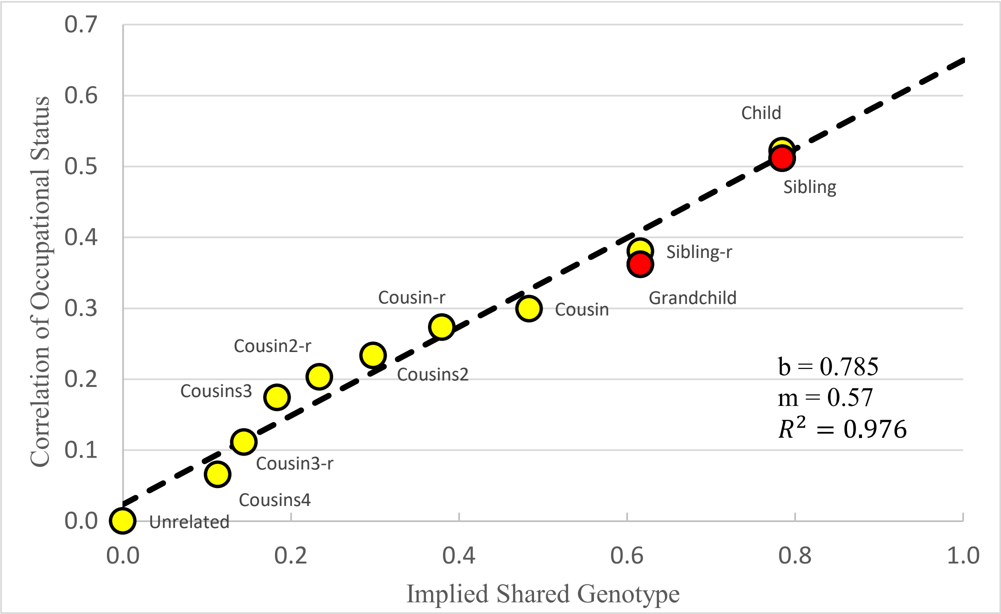

Figure S5: Occupational Status Correlations, births 1860-1919, and Implied Shared Genotype

Notes: The dashed line shows the OLS fit to this data. The R^2 reported is for this fitted line. The child and grandchild correlations potentially deviate from this fitted line.

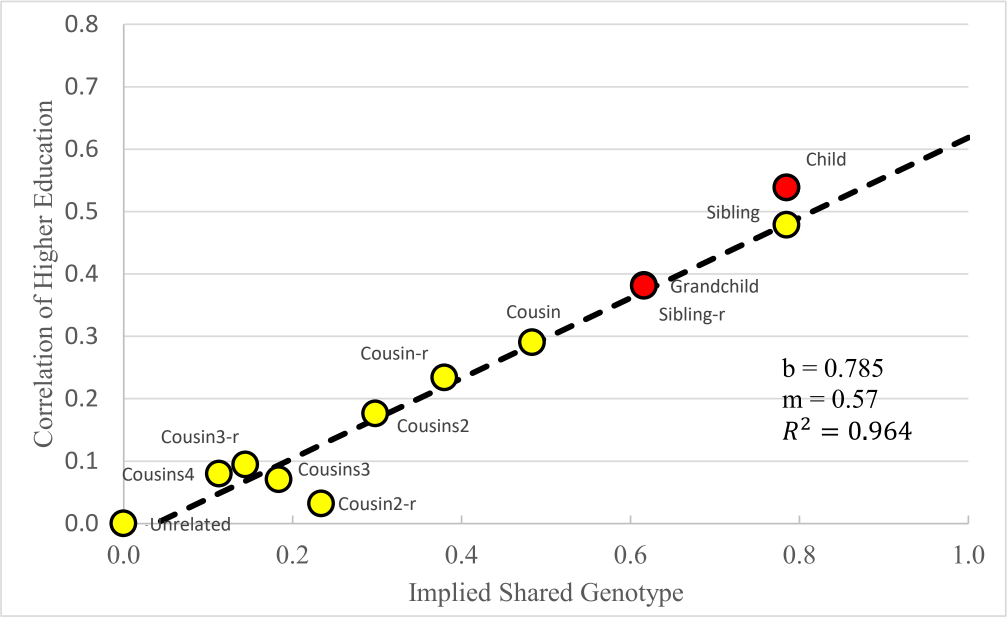

Figure S6: Higher Education Correlations, births 1780-1859, and Implied Shared Genotype

Notes: The dashed line shows the OLS fit to this data. The R^2 reported is for this fitted line. The child and grandchild correlations potentially deviate from this fitted line.

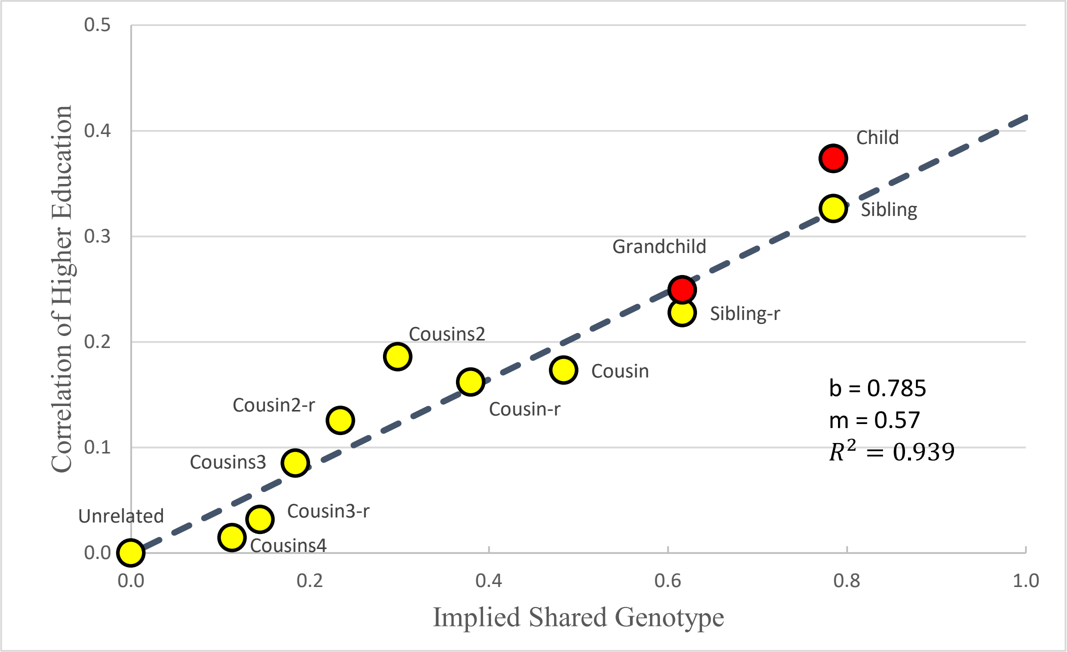

Figure S7: Higher Education Correlations, births 1860-1919, and Implied Shared Genotype

Notes: The dashed line shows the OLS fit to this data. The R^2 reported is for this fitted line. The child and grandchild correlations potentially deviate from this fitted line.

2. Marital Assortment

Table 4 reports the details of the estimate of the correlation, r, in latent social status between marital partners by period for marriages 1837-2022. In total there were 1,014,299 marriages with information on the occupational status of groom, father, and father-in-law. Bride occupational status was not used to estimate r because for most of this interval only a small minority of brides had a listed occupation.

We can estimate latent marital assortment for the years before 1889 using measures of bride literacy, indicated by the bride signing the marriage register. This estimation is just the ratio of the correlation of bride literacy to her father-in-law relative to the correlation with her own father. This gives similar results to those reported in table 5.[1]

Occupational status before 1940 was assigned using the HISCAM-GB scale.[2] Occupational status for 1940-2022 was assigned using the CAMSIS 1991 scale for Britain.[3]

3. The FOE Database

The materials for this study are a database of 422,374 individuals linked to their parents and spouses who lived, or had ancestors living, in England and Wales 1600-1822 (the Families of England (FOE) database). This database has two components. The majority of the data is from a set of lineages of persons with rare surnames created by members of the *Guild of One-Name Studies*.[4] These lineages incorporate everyone with a rare surname of interest, wherever they reside, as well as spelling variants of the surname. Thus the Mitchelmore lineage, for example, incorporates the surnames Michelmore, Mitchelmore, Mitchamore, Mitchmore, Mouchemore, Muchamore, and Muchmore.[5] Similary the Auty lineage encompasses Auty, Autey, Awty, Otty, and Ottey.[6] In cases where we only had access to the published lineages, these did not typically contain details of any living holders of the surname. In these cases we added that information ourselves from public records of births, marriages and addresses. Lineages were chosen for inclusion based on their completeness, and either the public posting of the lineages, or their creators' willingness to share the data with us for inclusion in the study.

In addition to these existing lineages, we ourselves created a set of lineages for rare surnames that were high wealth for people in the lineage born 1780-1850, for the purposes of better estimating social mobility rates through having more variance in social outcomes in the earlier generations. For the estimates based on the residence addresses in the electoral rolls and on company directorships of people in 1999-2022 we employ all persons in the lineages where the common ancestor was born 1780 and later. For estimates looking at educational and occupational status for people born 1780-1859 and 1860-1919 we exclude the high status lineages 1780-1850, because this will bias the estimates of persistence for relatives with ancestors born earlier than 1780. For literacy from marriage records for people marrying 1754-1879 we used again just the non-selected rare surname lineages.

Table S3: Families of England Data Outline

Birth Period	All	General Lineages	Elite Lineages
1600-99	5,720	5,472	248
1700-99	27,033	22,422	4,611
1800-49	62,366	52,373	9,993
1850-99	110,882	96,171	14,711
1900-49	76,167	67,485	8,682
1950-2022	46,554	40,556	5,998
All	421,907	363,685	58,222

Source: FOE database.

Table S4: Families of England, Numbers of Relationship Pairs

Relationship Pairs	All	General Lineages	Elite Lineages
Spouse	268,957	228,584	40,373
Child-Parent	495,153	431,292	63,861
Sibling (full)	508,144	441,024	67,120
Nephew/Niece	1,029,789	902,283	127,506
Grandchild	465,618	402,834	62,784
Cousin	804,009	726,359	77,650
Cousin Removed	1,713,957	1,563,495	150,462
2nd cousins	1,152,227	1,064,445	87,782
3rd cousins removed	2,345,628	2,204,766	140,862
3rd cousins	1,425,933	1,359,491	66,442
3rd cousins removed	2,628,002	2,531,016	96,986
4th cousins	1,464,685	1,424,859	39,826

Source: FOE database.

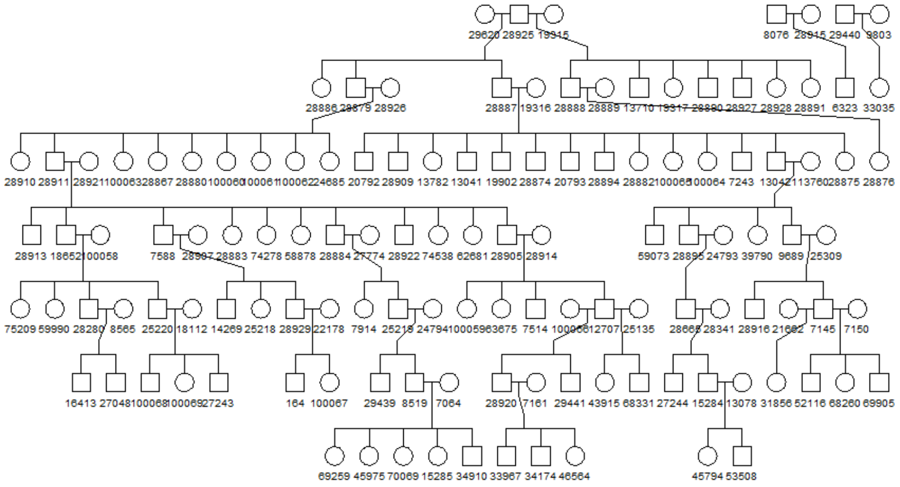

Figure S8: Sample of the FOE database

Notes: A sample section of the FOE database, showing linkages across 7 generations. The squares denote men, the circles women.

Table S3 shows the outline of the source of the data, and its distribution across time, and between general and elite lineages. Table S4 shows the numbers of relationship pairs in the data, again by lineage type. The reason for the extraordinarily large numbers of pairs of 2nd-4th cousins in table S4 can be seen in figure S8, which shows an illustrative fragment of the genealogy database. Average completed family size in England in the nineteenth century was around 3 adult children, but this varied enormously across families, and the bulk of adults in each generation came from larger than average families, so that average sibship size then was 6. Such demographic processes ensured large numbers of cousins, 2nd cousins, etc. for adults in each subsequent generation.

Table S5 shows the social outcomes that are available by gender and lineage type. The numbers of any social outcome are much less than the numbers of people in the database because: (1) some outcomes are available for men only, (2) before 1914 a significant number of children die before reaching age 21, (3) for births before 1780 and after 1920 many social outcomes are not observable. But, as table S3 shows, when we consider the numerous pairs of relatives in table S4, there are many pairs observed with the same social outcome, that can be used to estimate the underlying parameters in equation (3). Thus for male occupational status, for men born 1860-1919 the database contains 241,941 pairs of observations.

Table A5: Families of England, Social Outcomes

Outcome	Gender	All	General Lineages	Elite Lineages
Literacy	Both	6,700	6,700	0
Higher Education	Male	59,331	42,988	16,343
Occupational Status	Male	55,208	44,186	11,022
At Work 11-19	Both	31,961	26,891	5,070
At School 11-19	Both	31,961	26,891	5,070
Wealth at Death	Both	76,399	55,141	21,258
House Value	Both	24,746	19,864	4,882
Index of Multiple Deprivation	Both	24,898	19,958	4,940
Company Director	Both	32,973	27,112	5,861

Source: FOE database.

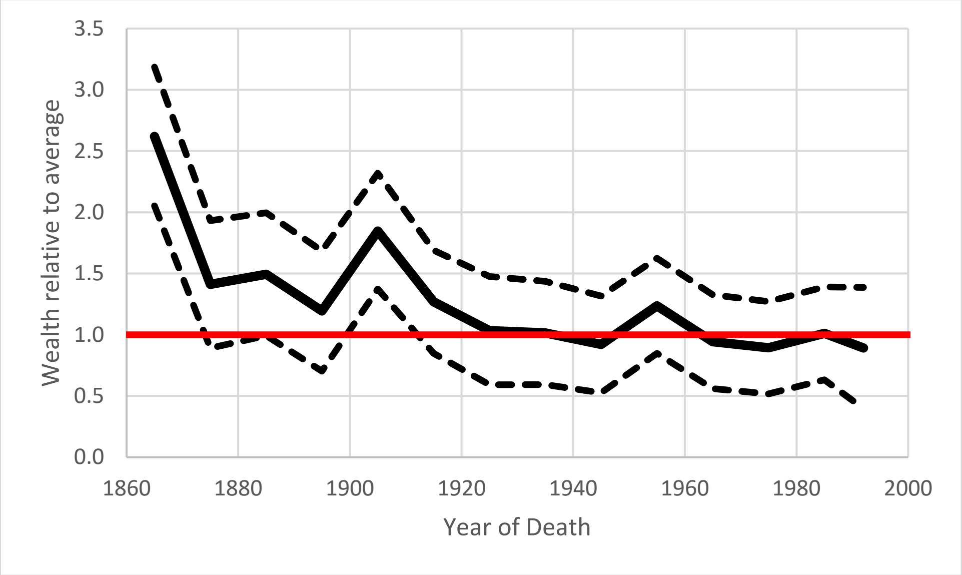

Figure S9: Wealth at Death, Average FOE Lineages relative to England

Notes: Average wealth at death by decade of members of the average FOE lineages relative to England and Wales as a whole. Dashed lines show 95% confidence intervals.

How representative are the lineages in the FOE database of the general population in England and Wales? One test is average wealth at death in the general lineage 1858-1996 compared to average wealth of all deaths in England and Wales in these same years. Figure S9 shows this ratio by decade 1860-1990. As can be seen for the death decades 1920 and later, and thus the birth decades 1860 and later, the Families of England average lineages seem representative of the general population in terms of wealth, and thus also in terms of other aspects of social status. For deaths before 1920, and thus births before 1860, average wealth in the general lineages is typically 50% higher than for the general population. The most likely explanation for this is that the processes that generated the rarer surnames used in these lineages were associated with somewhat higher status families in earlier centuries, but that over time slow but steady social mobility has brought these surnames to average social status by the time of births in the 1860s and later. There is also a possibility that lower status holders of the lineage surnames are less likely to appear in the records with a surname recognizable as belonging to these lineages.

Thus for the estimates of familial correlations using the average lineages for births 1860-1919 the sample used is then representative of the general population of England and Wales in its social outcomes. This conclusion is buttressed when we compare the average occupational status of men in the general lineages in the FOE database with the general population. For 16,639 men born in the FOE database who married 1880-1939, the average occupational status for average lineages was 37.2 on a scale 0-100, for those marrying outside the major city London. For grooms in a random sample of 591,000 Church marriages 1880-1939, transcribed by volunteers from the FreeReg organization, but drawn almost entirely from parishes outside London, the average occupation status on the same ranking was 35.5.[7] Thus again for births after 1860 the FOE sample looks very similar in status to the general population.

For men born 1780-1859 we can compare those in the FOE general lineages with the Freereg sample in terms both of occupational status, and of literacy. Men in the FOE general lineages marrying outside London 1837-79 had an average occupational score of 36.9. In contrast 771,000 grooms in the Freereg sample in these same years had an occupational score of only 31.1. Thus in line with the average wealth evidence, men in the average lineages scored nearly 20% higher than the average man in this period. The marriage records, also record if men and women can sign their names at marriage. In the Freereg sample of 484,888 marriages 1837-79 with records on whether brides and grooms signed, 68% of men and 58% of women were literate. For men and women in the FOE general lineages marrying outside London 1837-79, 73% of men and 58% of women were literate. Here the members of the FOE average lineages show only a very modest elevation in social status compared to the average person in England.

Will the modestly elite status of the FOE average lineages for births 1780-1859 bias the estimates of social mobility rates in this period? The answer is that if we draw a sample from the population where the variance of outcomes is different than for the population as a whole we would potentially get a biased estimate of the heritability of traits, h^2. If the variance of the sample outcomes is higher, we will also get a more precise estimate of persistence. But whatever sample of the population we start from, estimates of the level of persistence should be unaffected.

Table S6: FOE Observations 1999-2022 versus General Population

Region	National Population Share 2020[8]	FOE Address Data	FOE Address Share	National Average Dwelling Value 2017[9]	Average FOE Dwelling Value
London	0.151	1,914	0.078	£481,556	£576,957
East and South East	0.257	7,872	0.321	£306,534	£307,754
South West	0.096	2,883	0.118	£246,519	£254,057
Midlands	0.181	4,722	0.193	£183,773	£171,814
North	0.262	6,413	0.262	£152,318	£163,603
England	0.946	23,804	0.972	£240,325	£254,118
Wales	0.054	688	0.028	£151,672	£149,206

Source: FOE database.

For the recent period 1999-2022, where we observe house values, the Index of Multiple Deprivation, and Company Directorships table S6 shows the distribution of the observations used here, in terms of geography and house values versus for the general population. The FOE dataset for individuals observed 1999-2022 has a geographic distribution that largely echoes that of the general population distribution, except for being less frequent in London. But the FOE dataset is composed, by design, of long-established English family lineages. London is the area of England with the largest proportion of the population of more recent immigrants. So it is expected that the frequency of the FOE families will be lower in London than for the general population.

The estimated house values observed in the FOE database, adjusted to 2017 prices, again are close to the average across region observed nationally in sales in 2017. The only location with a substantial difference is London, where the FOE house values are higher. But as noted London is the city with the largest share of population of recent immigration to England. Thus there is no reason to expect the FOE house values here to be similar to those of the London population as a whole. Overall house values in the FOE database are 6.6% higher than for England and Wales, as a whole, in 2017. This is a modest difference.

The paper utilizes seven social outcome variables. These were constructed as follows.

1. Literacy. This is inferred for marriages 1754-1889 from whether the bride or groom signed the marriage register. These signature records extend more recently than 1889, but by then signature rates were very high, making the status information content of the measure low. Only a subset of county record offices in England have made images of marriage registers available on-line or through Ancestry.com. So literacy is available only for a subset of men and women marrying in these years.

2. Higher education. This is an indicator variable with a value 1 if the person achieved a higher educational status. Complete records are available for attendees Oxford and Cambridge Universities (1600-2018), Durham University (1837-1939), University of London (1837-1939), the Royal Military Academy Woolwich (1790-1839) and the Royal Military College Sandhurst (1800-1946). Complete records are available for the UK Medical Registers, 1859-2017, UK, Civil Engineer Lists, 1818-1930, UK, Electrical Engineer Lists, 1871-1930, UK, Mechanical Engineer Records, 1847-1930, UK, Articles of Clerkship (attorneys), 1756-1874. This variable is constructed for men born 1600-1919. Many of these records for the years before 1940 are available on Ancestry.com.

In the correlation estimations, correlations are calculated for all pairs of relatives where at least one member of the pair was born 1780-1859 or 1860-1919. To avoid having the outcome variable being measured in completely different epochs for a family pair, pairs were utilized only where they were no more than one generation apart, except for grandfather-grandson.

3. Occupational Status. Occupations are recorded in the censuses of 1841-1911, the population register of 1939, marriage records 1837-2022, probate records, 1858-1939, ship passenger lists, 1870-1959, army enlistment records 1914-1918, and in the professional directories listed above. Where a person had multiple statements of occupation at different times in the life course, the one closest to age 40 was employed. For those born 1780-1919 the very large set of occupation description strings were first assigned to one of 442 categories. Using a large set of 1.6 million marriage records 1837-1939 which give occupations for grooms, their fathers, and the father-in-law an occupational status score 0-100 was derived using Goodman's Association methodology. As can be seen in table 2, the occupational status index derived in this way shows strong parent-child correlations in both 1780-1859 and 1860-1919.[10]

4. House Value. This is estimated from the addresses recorded for people alive 1999 and later in the electoral roll. Since the Roll released 2002 and later records only those who consented to the release of their address, there are potential issues of selectivity. However, we see in table S6 above that the average house value recorded using the electoral register addresses closely approximates to that of England as a whole. There is thus no sign that higher status individuals are less likely to permit publication of their addresses in the Electoral Roll.

The Land registry shows house prices for all property sales 1995 and later. From this we construct an average dwelling value, normed to 2017 prices, for each Postal Code. British Postal codes on average cover only 40 houses. So this gives a good estimate of local house values for the person. Where there was no sale recorded for a postcode, we use the Council Tax Band to

estimate the property value. Empirically the log of average house values produces higher correlations between relatives, so we use this measure.

We employ this measure only for men and women aged 24 or above, and living at a different address than their parents.

Property values differ substantially by region in England and Wales, as table S6 shows. London house values, for example, are more than 4 times those in the North of England. Since people show strong persistence by region across generations, we normalize all house values to remove regional effects, dividing England and Wales into 6 regions for this purpose.

5. Index of Multiple Deprivation. This index is a ranking of Lower Layer Super Output Areas (LSOAs), typically with a population of around 1500, in terms of a weighted average of measures of social deprivation. The index is available by post code.[11] The 2019 index, used here, is a weighted average, with weights indicated, of measures of: Income (22.5%), Employment (22.5%), Education (13.5%), Health (13.5%), Crime (9.3%), Barriers to Housing and Services (9.3%), Living Environment (9.3%). Since the index for Wales is constructed in a non-comparable way, we fix the level for that as the average for the English IMD for the North of England.

6. Company Director. Companies House in the UK maintains a register of the directors of limited companies.[12] Limited companies include commercial enterprises, but also management companies for housing associations, as well as medical and legal practices. The register also includes people who subsequently resigned the position. We classify anyone alive 2002 and later, and aged 24 and above in 2022, as either a company director or not.

7. Status Modern. This is an index of social status which combines the three previous measures, using Principal Component Analysis, into an omnibus modern social status index. The correlation of the normed log house value and the index of multiple deprivation is 0.53, and with being a company director 0.24. The correlation between the index of multiple deprivation and with being a company director is 0.14. The correlation of the Status Modern index with these three components is: normed log house value, 0.86, index of multiple deprivation, 0.82, company director, 0.49.

4. Replication Files

Included in the supporting information are two Excel files with the data required to replicate the results in Figures 1-4, S1-S7, and in tables 2-4, and S2.

The file Clark-Replication Data contains

(a) the details for the 11 sets of relatives used to calculate the correlations for births in the period 1910-97 in table 2, and portrayed in Figures 2, and S1-S3.

(b) the details for the 11 sets of relatives used to calculate the correlations for births in the period 1754-1919 in table 2, and portrayed in Figures 2, and S4-S7.

(c) the details of occupational status for groom, father and father-in-law for a set of marriages in England and Wales 1837-2021, used to calculate implied phenotype assortment in marriage.

(d) the data underlying the correlations portrayed in figure 3, and the regression coefficient estimates portrayed in figure 4.

The file Clark-FOE Raw Data contains all the persons in the FOE database born 1600 and later, with the links to their father and mother, and with the 7 social outcomes used in this study. From this file the 11 relationship combinations and their status outcomes can be constructed.

References

1. G Clark, N. Cummins, Assortative Mating and the Industrial Revolution: England, 1754-2021. (2022). https://hub.cepr.org/discussion-paper/138817.
2. Paul S. Lambert, et al. The Construction of HISCAM: A Stratification Scale Based on Social Interactions for Historical Comparative Research, *Historical Methods: A Journal of Quantitative and Interdisciplinary History*, 46:2, 77-89, (2013) DOI: 10.1080/01615440.2012.715569
3. Prandy, K., and Lambert, P. S. Marriage, Social Distance and the Social Space: An alternative derivation and validation of the Cambridge Scale. *Sociology*, 37(3), 397-411. (2003)
4. The Guild of One-Name Studies [internet] [cited 2023 April 4]. Available from: https://one-name.org/
5. M*ch*more One Name Study [internet] [cited 2023 April 4] Available from: https://mitchelmore.one-name.net/
6. Auty One-Name Study [internet] [cited 2023 April 4] Available from: http://auty-1.one-name.net/
7. Free UK Genealogy "FreeREG" [internet] [cited 2023 April 4] Available from: https://www.freereg.org.uk
8. Statistica: Population of the United Kingdom in 2021, by region. [internet] [cited 2023 April 4] Available from: https://www.statista.com/statistics/294729/uk-population-by-region/
9. HM Land Registry, UK House Price Index (HPI) for June 2017 (8/15/2017) [internet] [cited 2023 April 4] Available from: https://www.gov.uk/government/news/uk-house-price-index-hpi-for-june-2017
10. Clark, G, N Cummins, and M Curtis. The Mismeasurement of Man: Why Intergenerational Mobility is much lower than Conventionally Measured, England, 1800-2021. (2022). https://cepr.org/publications/dp17346.
11. UK Ministry of Housing, Communities and Local Government. English Indices of Deprivation 2019. 9/26/2019 [internet] [cited 2023 April 4]. Available from: https://www.gov.uk/government/statistics/english-indices-of-deprivation-2019
12. UK Companies House Register of UK Company Directors [internet] [cited 2023 April 4] https://find-and-update.company-information.service.gov.uk/
