# Supplementary material for: The inheritance of social status: England, 1600 to 2022
Source: Proc Natl Acad Sci U S A. 2023 Jun 26;120(27):e2300926120. doi: 10.1073/pnas.2300926120 (PMC10319028; doi:10.1073/pnas.2300926120)
Supplement: Supplementary file 2 — Appendix 02 (PDF) [file pnas.2300926120.sapp2.pdf]

Assortative Mating and the Industrial Revolution: England, 1754-2021

Gregory Clark, UC Davis, LSE and CEPR[1]

Neil Cummins, LSE and CEPR

Using a new database of 1.7 million marriage records for England 1837-2021 we estimate assortment by occupational status in marriage, and the intergenerational correlation of occupational status. We find the underlying correlations of status groom-bride, and father-son, are remarkably high: 0.8 and 0.9 respectively. These correlations are unchanged 1837-2021. There is evidence this strong matching extends back to at least 1754. Even before formal education and occupations for women, grooms and brides matched tightly on educational and occupational abilities. We show further that women contributed as much as men to important child outcomes. This implies strong marital sorting substantially increased the variance of social abilities in England. Pre-industrial marital systems typically involved much less marital sorting. Thus the development of assortative marriage may play a role in the location and timing of the Industrial Revolution, through its effect on the supply of those with upper-tail abilities.

Marital assortment is important both for rates of intergenerational social mobility, and for the distribution of abilities within society in any generation. The more assortment, the greater will be social inequalities within generations, and the slower rates of social mobility. It has also been widely assumed that the rise of women's education and employment outside the home led to greater degrees of marital assortment since at least the 1940s.[2] This would imply earlier societies had more social mobility, and lower inequalities in social abilities and outcomes, than for current generations.[3]

---

[1] The substantial data collection embodied in this paper was made possible by generous financial support for Economic History at UC-Davis from Michael Dearing.

[2] See Mare, 1991, for example, which claims an increase in the strength of educational assortment in the USA 1940-1987.

[3] This is the dystopia envisaged by Young (1958).

There has been, however, surprisingly little study of the degree of marital assortment over the long run. This stems from the fact that until the late nineteenth century women had few formal educational qualifications. Also many women did not have formal occupations, even before marriage. Many were confined to domestic roles, particularly in upper class families.

In this paper we show how marriage certificates in England, unchanged since 1837, can be used to estimate the degree of matching in underlying occupational and educational abilities between grooms and brides, independent of measurement errors, even where there is no direct measure of bride occupational status or educational ability.[4]

These measures show three surprising things. The first is that there is no sign of any substantial increase is marital assortment by education or occupational status in England 1837-2020. The second is that marital assortment is much closer than conventionally measured. For England the conventionally measured correlation of occupational status between husband and wife in terms of occupation at marriage was 0.39, but the measure we develop below implies a correlation in the same period of 0.8. The third is that the underlying rate of intergenerational correlation in occupational status is also high throughout the years 1837-2021.

The more limited information from marriage registers 1754-1836 on bride and groom literacy suggests that the strong assortment in marriage observed from 1837 on was also found 1754-1836.

We show below that the high degree of marital assortment we find will substantially increase the population variance in occupational and educational abilities. We show also that any change in the degree of assortment in marriage will take a substantial number of generations to create this increase in population variance. Thus changes in marital patterns in the late middle ages in Europe would potentially affect the population distribution of abilities fully only 500 years later at the time of the Industrial Revolution. We show also that earlier marriage systems where families arranged the match would typically display much less assortment, and thus less variance in the population of social abilities.

[4] For marital assortment we are using here a method that was introduced by Curtis (2021). The Curtis paper finds similar evidence of strong marital assortment in Quebec, 1800-1970.

Marital Assortment, Social Mobility and Inequality

As noted, there should be a connection between marital assortment and intergenerational mobility. Suppose x is underlying occupational or educational abilities. In fathers this will be always manifest in an occupation or educational outcome, while in earlier years for mothers there will be no occupation outside the home and no formal educational outcome. Suppose also that occupational abilities are inherited equally from fathers and mothers, as the empirical evidence below will suggest. Then

$$x_c = \frac{1}{2}\beta\left(x_f + x_m\right) + e \tag{1}$$

where c, f, and m indicate child, father and mother, and β is the intergenerational correlation in occupational abilities between the average of the parents and the child.[5] If the correlation between parents in occupational abilities is r, then

$$x_f = r x_m + v \tag{2}$$

This means that the intergenerational correlation between a child and a single parent in occupational abilities will be

$$b = \beta\frac{(1+r)}{2} \tag{3}$$

Thus the greater is assortment, the lower will be social mobility rates. The evidence from English marriages 1837-2021 below suggests that r = 0.8, b = 0.9. This would in turn imply from (3) that $\beta = 1$.[6]

From (1) the variance of x_c will be

$$\sigma_{x_c}^2 = \frac{1}{4}\beta^2 var\left(x_f\right) + \frac{1}{4}\beta^2 var(x_m) + \frac{1}{2}\beta^2 cov\left(x_f x_m\right) + \sigma_e^2 \tag{4}$$

[5] This specification assumes perfect substitutability between parents in the transmission of abilities to children. Some may object that parents could be complementary in child outcomes. In table 6 we validate the substitutability assumption empirically. If parents were complementary in this way it would magnify the effect of marital assortment on the variance of child outcomes.

[6] $\beta = 1$ implies children fully inherit the underlying occupational abilities of parents.

In long run social equilibrium, σ_x^2 will be the same across generations, and across men and women. Since $var(x_f) = var(x_m) = \sigma_x^2$, and $cov(x_f x_m) = r\sigma_x^2$, where r is the correlation between x_f and x_m, then (1) implies that in equilibrium

$$\sigma_x^2 = \frac{1}{2}\beta^2\sigma_x^2 + \frac{1}{2}r\beta^2\sigma_x^2 + \sigma_e^2$$

$$\Rightarrow \sigma_x^2 = \frac{\sigma_e^2}{1-\frac{1}{2}\beta^2(1+r)} \quad (5)$$

With β = 1, as we observe for England 1837 and later, (5) becomes

$$\sigma_x^2 = \frac{\sigma_e^2}{1-\frac{(1+r)}{2}} \quad (6)$$

This implies that the variance of social abilities will depend strongly on the degree of assortative mating. If r increases from 0 to 0.8, then the variance of underlying abilities under (6) would increase by a factor of 5. Figure 1 shows the implied equilibrium distribution of social abilities under (6) with no marital assortment versus a 0.8 correlation between parents.

This in turn implies an enormous increase in the proportion of the population at the tails of the distribution. With no marital assortment the share of the population in the top 2.5% of abilities with a marital correlation of 0.8 would be 0.0006%. Even a switch from a 0.8 correlation to a 0.5 correlation would mean only 0.16% of the population would have occupational abilities in the top 2.5% for the 0.8 correlation, less than one fifteenth as many.

Note, however, that if the degree of assortment increases in marriage then it will take multiple generations for this to have a full effect on the population distribution of abilities. If we rewrite (5) in terms of the dynamics of σ_x^2 over generations, indexed by t, then

$$\sigma_{x_{t+1}}^2 = \beta^2 \frac{1+r}{2} \sigma_{x_t}^2 + \sigma_e^2 \quad (7)$$

Figure 1: Marital Assortment and the Social Distribution of Abilities

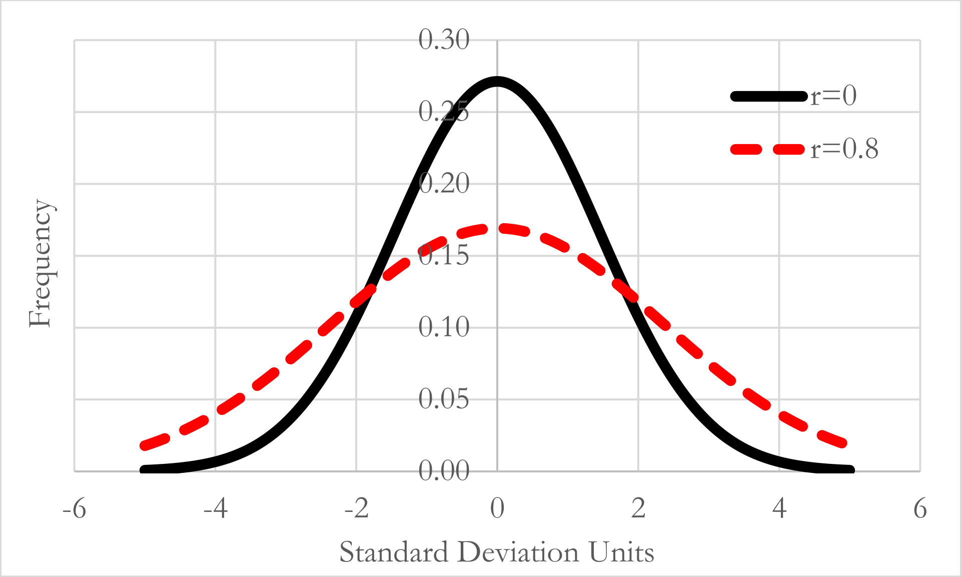

Note: The standard deviation units are for the situation of zero assortment in marriage.

Figure 2: Dynamics of an Increase in Marital Assortment

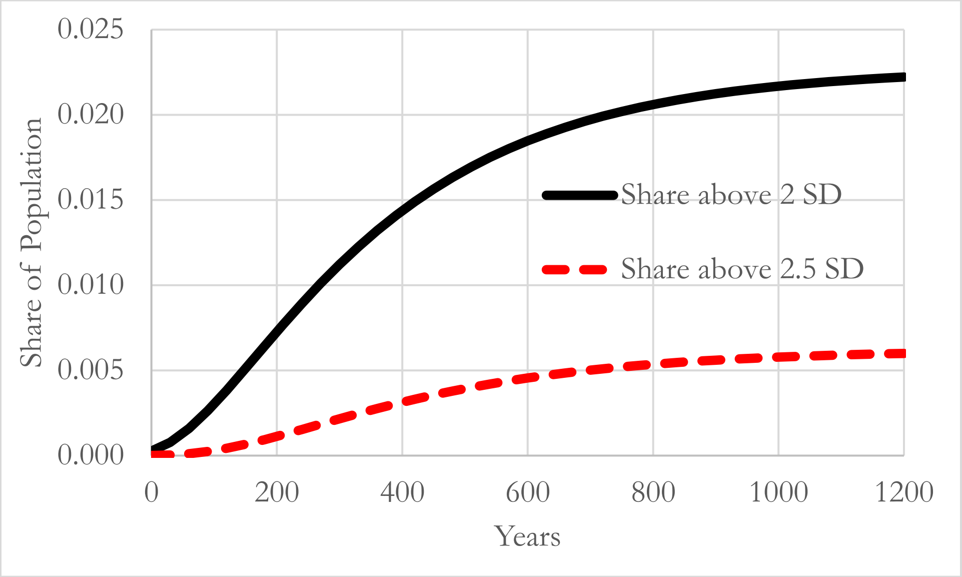

Notes: The figure shows the share of the population with abilities above 2 and 2.5 standard deviations above the mean in the equilibrium population with a marital correlation of 0.8 on social abilities, where the starting population has a 0.4 marital correlation.

Figure 2 shows the dynamics over time, counting a generation as 30 years, of a switch from a 0.4 correlation of status in marriage, to the 0.8 correlation observed for England marriages 1837-2021. The figure shows for each generation in the transition the share of the population which will have abilities above 2 or 2.5 standard deviations of the new equilibrium distribution. Note that it takes 300 years for the share of the population above 2 standard deviations from the mean to reach half its new equilibrium level, and 510 years to reach three quarters of the new level.

Measuring Assortment and Intergenerational Persistence

In measuring marital assortment we have information on the occupational status of grooms, their fathers, and their fathers-in-law 1837-2021. We also have measures of bride's occupational status in some cases 1837-2021, though on a different occupational status scale to men. Finally, we have measures of groom and bride literacy 1837-1879.[7]

There are three issues in measuring marital assortment over the long run. The first is that even if we had consistent measures of occupational status for husbands and wives over time, these measures all contain considerable error. A term such as "clerk," for example, covers people of a whole variety of actual occupational status. And the average status assigned to this loose status label will itself contain some error.

Second, over time as occupational structures and titles changed the errors associated with assigning status to occupations also potentially changed. Studies that try to estimate from occupational status data differences in the degree of intergenerational mobility over time, such as Long and Ferrie, 2013, Perez, 2019 and Xi et al., 2020, or of marital assortment, across time and across countries, will be vulnerable to changes in the degree of measurement error across time and place.

Third, most women did not have formal educational attainments, or formal occupations, until the twentieth century in England. Also in the marriage records there was selective

[7] These measures extend beyond 1879, but by then literacy was so common that there is little information on assortment in the literacy record.

reporting of women's occupations at different times. In the nineteenth century daughters of higher status fathers were less likely to report an occupation at marriage than daughters of lower status fathers. By 1980 women with higher status fathers were more likely to record an occupation. So how can we measure in a consistent way the degree of correlation in attributes between men and women in marriage?

Here we show that we can use the occupational status of fathers as a way of estimating, independent of these measurement errors, both the degree of assortment in marriage, and the intergenerational correlation of status. Let y_f and y_m be the observed status of men and women respectively, and, as above, x underlying status, where

$$y_f = x_f + u_f \qquad (8a)$$

$$y_m = x_m + u_m \qquad (8b)$$

and u denotes the error term. Note that because of gender differences in occupations this error term will potentially be different for men and women.

In this case, as the first panel of figure 3 shows, the correlation in underlying status between the groom and father-in-law will be rb, while that between father and father-in-law will be rb^2.

Since all outcomes are observed with measurement error, however, as in (8a) and (8b) the observed correlations will be attenuated by those errors. If we assume the error potentially has a different magnitude for male and female occupations, then the pattern of attenuation from measurement errors will be as in the second panel of figure 3. Where the correlation is measured between males, the attenuation will be a factor θ. But where the correlation is between occupations for men and women, the attenuation factor will be potentially a different factor ϕ. Again the second panel of figure 3 shows the pattern of attenuation in the observed correlations.

Figure 3 also shows, however, that we can get error independent measures of r and b by taking the ratio of correlations. Thus the underlying matching correlation in marriage r can be estimated from

$$r = \frac{\rho_{groom-father-in-law}}{\rho_{groom-father}} = \frac{\theta rb}{\theta b} = \frac{\rho_{bride-father-in-law}}{\rho_{bride-father}} = \frac{\phi rb}{\phi b} \tag{9}$$

Figure 3: Implied Correlation Structures if Assortment is by Husband-Wife

A. Underlying Correlations

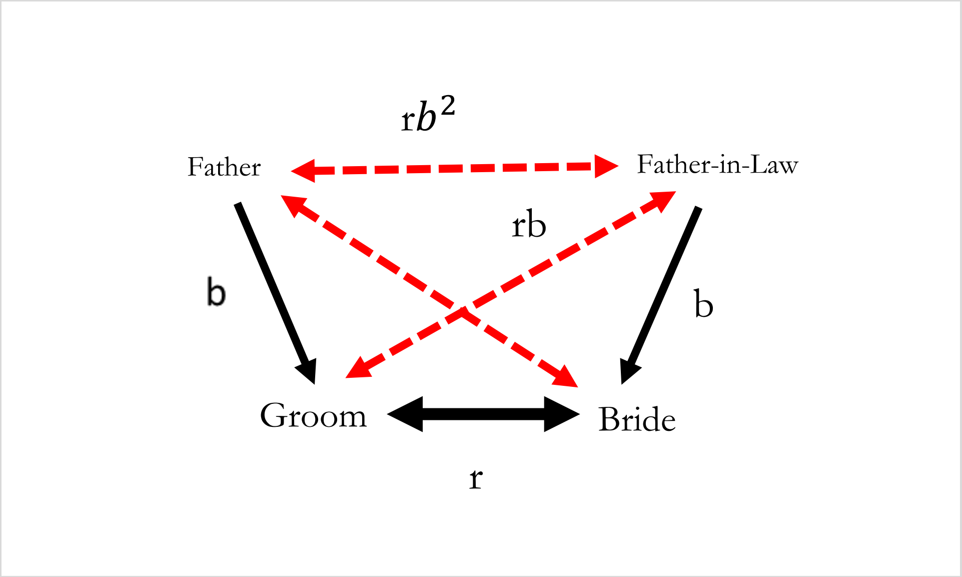

B Observed Correlations

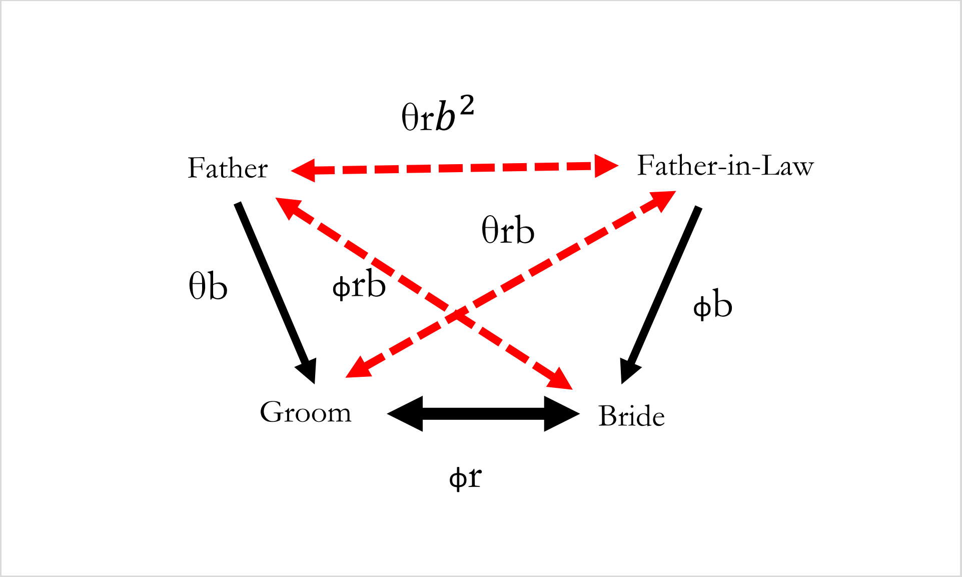

Note: Causal correlations are indicated by black lines, and non-causal ones by red.

This estimate of matching in marriage depends on the correlation in "potential" occupational status of brides being the same with their fathers as the correlation of groom and father. But, as noted, we show evidence for exactly this assumption below.

Note, however, that the selective sample of bride occupations will potentially bias the estimate of assortment through the bride correlations. The advantage of this estimate for looking at degrees of marital assortment across long periods is that changes in θ and ϕ, the attenuation of observed correlations from the looseness of occupational scales or other measures of status, will have no effect on the estimate of r.

The underlying intergenerational correlation similarly will be

$$b = \frac{\rho_{father-father-in-law}}{\rho_{groom-father-in-law}} = \frac{\theta r b^2}{\theta r b} = \frac{\rho_{bride-father-in-law}}{\rho_{bride-groom}} = \frac{\phi r b}{\phi r} \quad (10)$$

This estimate again depends of the symmetry of inheritance of characteristics by groom and bride. Again the selective sample of bride occupations will potentially bias the estimate of intergenerational correlations through the bride correlations.

Another way to estimate b, evident from figure 3, is to use father-in-law occupational status, which is correlated both with the groom's status and the groom's father's status, as an instrument in estimating the underlying father-son correlation. This should produce an estimate of b that is the same as from expression (10), if the symmetry assumption holds.

For the years before 1880 we also have universal evidence on bride and groom literacy, from their ability to sign their names on the marriage record as opposed to making a mark. We can use this evidence to test the assumption above of a symmetrical transmission of status from fathers to daughters versus sons. In figure 4 we allow for a different potential strength of correlation between father occupational status and child literacy for sons versus daughters, b_m versus b_f. Now if we look at the correlations between literacy of the bride and groom and occupational status of the fathers then we will get the pattern of observed correlations shown in figure 4. There are four different attenuation effects θ, ϕ, ψ, and λ, given that bride literacy rates are lower than groom rates. Now

Figure 4: Observed correlations with father occupations and groom/bride literacy

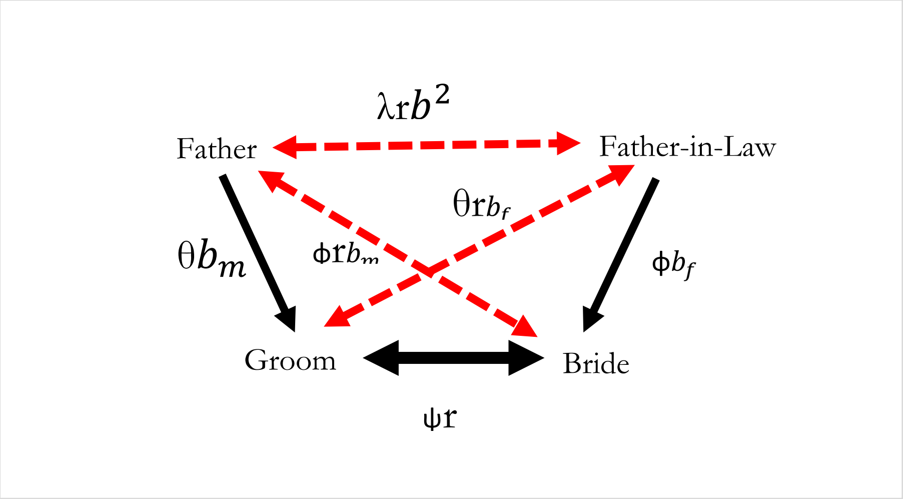

$$\frac{\rho_{groom-father-in-law}}{\rho_{groom-father}} = \frac{\theta r b_f}{\theta b_m} = r\frac{b_f}{b_m} \tag{11}$$

$$\frac{\rho_{bride-father-in-law}}{\rho_{bride-father}} = \frac{\phi r b_m}{\phi b_f} = r\frac{b_m}{b_f} \tag{12}$$

If $b_m = b_f$, then both (11) and (12) will provide the same value of r. But if $b_m \neq b_f$, then still

$$\frac{\rho_{groom-father-in-law}}{\rho_{groom-father}} \times \frac{\rho_{bride-father-in-law}}{\rho_{bride-father}} = \frac{\theta r b_f}{\theta b_m} \times \frac{\phi r b_m}{\phi b_f} = r^2 \tag{13}$$

Thus in the period 1837-1879 we can get an error independent estimate of marital assortment in education, which is independent of the assumption of symmetrical status transmission from fathers to sons and daughters.

The Symmetry Assumption

We assume in (9) and (10) above that the intergenerational correlation of actual occupational status for grooms with their fathers is the same as the intergenerational correlation of potential occupational status of women with their fathers. Women inherit occupational "abilities" as strongly as men, so that marital matching is symmetrical.

We suggest above a test of this assumption for earlier years using equations (11)-(12). However, we where we have information on social status for men across three generations - fathers, son, and grandsons – we can test again whether the symmetry assumption works. With complete symmetry men and women will correlate equally in social abilities with their fathers, and also with their sons. In that case grandsons will correlate equally with paternal and maternal grandfathers. In contrast, if the inheritance of abilities is lower for women relative to their fathers, and lower for sons relative to their mothers, then male grandchildren will correlate more closely with the paternal grandfather.

Thus if we run a regression including measures of the status of both the paternal and maternal grandfather, of the form

$$y_{gc} = a + b_p y_{pgf} + b_m y_{mgf} + e \quad (14)$$

where *gc* indicates grandchild, *pgf* paternal grandfather, and *mgf* maternal grandfather, then we should find $\hat{b}_p = \hat{b}_m$. The prediction on grandchild outcomes should be as strong from the maternal side as from the paternal.

Note that if we do find just such symmetry, then it also implies that a high degree of marital assortment will both increase the intergeneration correlation of social abilities, b, and also increase the society-wide variance of social abilities. If child outcomes depend only on the characteristics of the father, then marital assortment would have no effect on social mobility rates or the variance of abilities.

Data Sources

Marriage Registers, 1837-2021. From 1837-2021 a marriage certificate in England and Wales, whether the marriage was performed in a church or a registry office gives:

(1) marriage date and place
(2) names of the bride and the groom, their ages, their marital condition (single/widowed), their "rank or profession", and their residences at the time of the marriage
(3) names and "rank or profession" of their fathers
(4) signatures or marks of the bride, groom, and witnesses

Figure 5 shows examples of such a certificate, which has been used unchanged 1837-2022, from both 1837 and 2020.

The UK government now has such records of around 106 million marriages 1837-2022 from England and Wales, with the associated details. However, it costs by statute £11 to obtain a copy of any marriage certificate from the government, and the copy is delivered by mail as a paper reproduction of the government's copy of the marriage certificate. Since copies of the marriage certificates were kept in church registers, and many of these registers have since been deposited in local record offices, these provide an alternative source for marriage records.

The marriage certificates available in record offices exclude Civil Marriages. But though Civil marriage was introduced in England in 1837, such marriages remained a minority of all weddings before 1914. In 1841 Civil marriages were 1.7% of all marriages, and in 1914, still only 24%. Thereafter there were increasing numbers of civil weddings, as church attendance declined, but also as divorce rates increased. Until recently divorcees were rarely granted permission to be remarried in the Church of England. So 31% of weddings were civil by 1952, 49% by 1982 and 68% by 2012. However for first marriages by 1995 only 40% were civil, and for 2012 63% civil (Haskey, 2015).

Figure 5: The English Marriage Certificate 1837-2021

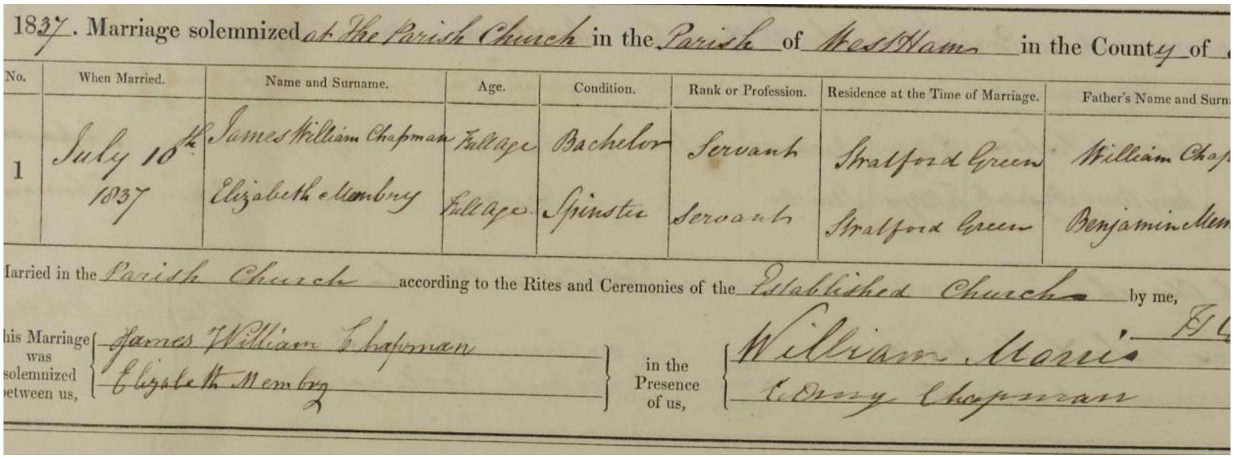

1837. Marriage solemnized at The Parish Church in the Parish of West Ham in the County of

No.	When Married.	Name and Surname.	Age.	Condition.	Rank or Profession.	Residence at the Time of Marriage.	Father's Name and Surn[ame]
1	July 10th 1837	James William Chapman	Full age	Bachelor	Servant	Stratford Green	William Chap[man]
		Elizabeth Membry	Full age	Spinster	Servant	Stratford Green	Benjamin Mem[bry]

[M]arried in the Parish Church according to the Rites and Ceremonies of the Established Church by me,

[T]his Marriage was solemnized [b]etween us, James William Chapman, Elizabeth Membry

in the Presence of us, William Morris, [illegible] Chapman

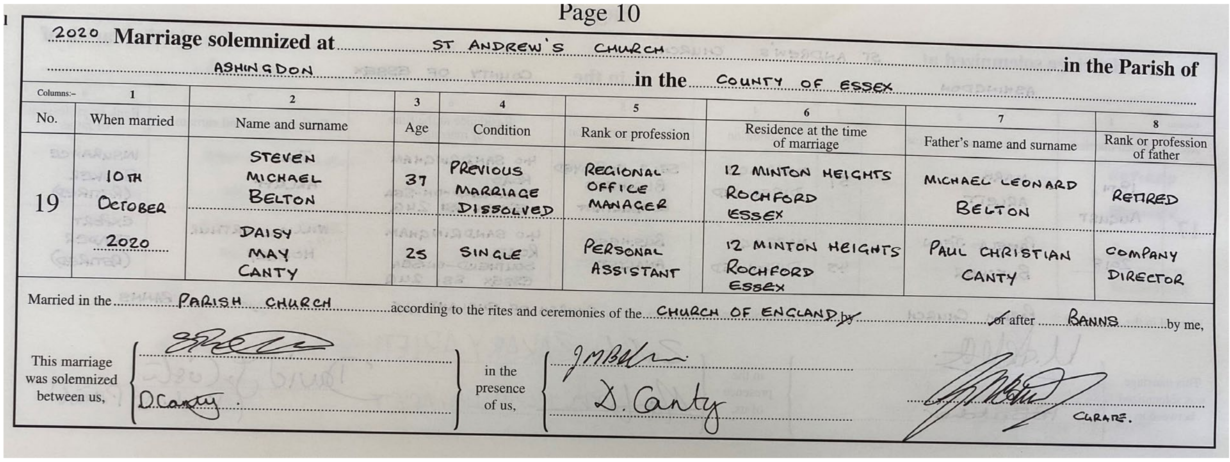

Page 10

2020 Marriage solemnized at ST ANDREW'S CHURCH in the Parish of ASHINGDON in the COUNTY OF ESSEX

No.	When married	Name and surname	Age	Condition	Rank or profession	Residence at the time of marriage	Father's name and surname	Rank or profession of father
19	10TH OCTOBER 2020	STEVEN MICHAEL BELTON	37	PREVIOUS MARRIAGE DISSOLVED	REGIONAL OFFICE MANAGER	12 MINTON HEIGHTS ROCHFORD ESSEX	MICHAEL LEONARD BELTON	RETIRED
		DAISY MAY CANTY	25	SINGLE	PERSONAL ASSISTANT	12 MINTON HEIGHTS ROCHFORD ESSEX	PAUL CHRISTIAN CANTY	COMPANY DIRECTOR

Married in the PARISH CHURCH according to the rites and ceremonies of the CHURCH OF ENGLAND by ~~or~~ after BANNS by me,

This marriage was solemnized between us, [illegible], D.Canty

in the presence of us, J M Belton, D. Canty

[illegible] CURATE.

Thus the available marriage records will give an unbiased estimate of marital assortment in the years before 1914. For more recent years there will be potential issues of positive or negative selection into church weddings. But while positive or negative selection might reduce the measured bride-groom and father-child correlations, selection will not necessarily affect the underlying correlations which are a ratio of two observed correlations.

Anglican church marriage records will also, until recently, generally exclude second marriages where one of the parties has a living spouse from a first marriage.[8] However, there is no reason to expect that assortment will be any different in first compared to subsequent marriages, or that men and women in first marriages have any different an occupational or educational correlation with their parents than those in subsequent marriages following divorce.

The Freereg Organization of genealogy volunteers has been digitizing and placing on the web marriage records for a number of years. From their web site we were able to collect 1.6 million marriage records in England 1837-2010.[9] However, because of the genealogical interests of its members, the Freereg volunteers mainly digitized the marriage records from before 1940. Thus for the years 1940-2021 we supplemented these records with a set of marriage records from Essex, where the traditional county of Essex conveniently includes both parts of what is now London, as well as rural areas. Thus for the years 1980-2021 we have 5,497 marriage records from the Freereg web pages, and an additional 7,845 records from our own collection from Essex. Tables 1 and 2 summarize the data we have available on marriages 1837-2021. [10]

Because transcribing these marriage records is a volunteer effort based on local interests, the numbers of marriages recorded by county for the years 1837-1940 vary considerably. Four counties contain about 50% of the marriages transcribed for England: Kent, Lancashire, Lincolnshire, and Staffordshire. But these counties were distinct from each other in terms of occupations and urbanization, so that the sample generated seems representative of England as a whole.

Note in table 1 that the amount of occupational information for women is much less than for men until after 1980. Many women had no listed occupation, or such non-informative occupations as "at home". And the women reporting no occupation are from high social status families in the early years, and then from lower status families in the later years.

[8] The proportion of all marriages involving someone who was divorced was less than 0.3% before 1914, because of the difficulty of obtaining divorce in these years.
[9] https://www.freereg.org.uk/
[10] There are 1,637,674 marriages 1837-2010 from Freereg. In addition there are 27,887 from 1837-2021 from Essex parishes, including some now part of London, we ourselves collected.

Table 1: Parish Register Marriage Data, 1837-2021

Marriage Period	All	Groom Occupation	Bride Occupation	Father Occupation	Father-in-law Occupation
1837-59	540,650	450,905	70,032	413,638	411,789
1860-79	365,465	310,321	42,146	294,935	295,259
1880-99	336,124	285,405	42,870	253,004	273,058
1900-39	343,344	283,040	63,397	242,408	273,831
1940-79	66,636	61,454	39,380	52,986	54,405
1980-2021	13,342	12,288	10,653	10,659	10,912
All	1,663,478	1,401,806	198,446	1,266,052	1,317,687

Table 2: Parish Register Literacy Data, 1837-1879

Marriage Period	All	Groom Literacy	Bride Literacy
1837-59	540,650	274,898	274,900
1860-79	365,465	90,473	90,472
All	906,115	365,371	365,372

If we take, for example, women marrying 1837-79 whose fathers had occupational status scores of 75 (out of 100) and higher, only 1% have an occupation listed. In contrast, in the same period for fathers with an occupational status score of less than 25, 12% of brides had occupations listed. This problem of the selective absence of female occupations is found all the way until 2021. But in the later years the tendency for absence switches towards women of lower socioeconomic status. Thus for marriages 1980-2021 daughters of fathers with occupational status above 75 report an occupation 95% of the time, but daughters of fathers with occupational status less than 25 report an occupation only 60% of the time. These selective omissions will bias the observed correlations between brides and grooms, fathers and fathers-in-law. In contrast, male occupations are universally reported, and will give unbiased estimates.

Table 2 shows the number of observations on groom and bride literacy 1837-1879. There is data on literacy for the years 1880 and later also, but with the introduction of general grade level education in 1872, literacy rates rose substantially thereafter so that the literacy evidence is not informative of educational levels of bride and groom for marriages after 1879. Only some transcribers for Freereg recorded whether brides and grooms were literate, so the information here is incomplete. However, there is little difference in the rate at which literacy is recorded for the marital partners for brides with high or low occupational status fathers. At the high end of occupational status literacy is reported for 53% of marriages, while at the low end of occupational status it is recorded for 58% of marriages. Thus the literacy evidence will provide an unbiased estimate of the degree of marital assortment.

Another feature of the marriage registers is that groom occupations will typically be recorded around age 25-30, while the fathers' occupations are recorded at ages 55-60. There is some occupational upgrading over the life course, so this will reduce the father-son occupational correlation below what would be observed if occupations for both were measured at age 40. But nicely any noise this adds to the estimates will be the same for the father-son correlation as for the father-in-law son correlation. However, we also observe that the measurement error for occupations is becoming greater over time. This will potentially bias the estimates of the intergenerational correlation from equation (6), since this is estimated as the ratio of an intergenerational correlation and a contemporaneous correlation.

To rank occupations we used the HISCAM occupational rankings for males in Britain 1800-1938 to assign occupational scores for marriages 1837-1939.[11] For marriages 1940-2021 we assigned occupational scores using the CAMSIS 1990 scores, which are based on a 1% sample of British households in the 1991 UK census.[12]

Families of England Database, 1700-2020. This database, with 412,636 persons, follows as far as possible the family connections of a set of families with rarer surnames in England, 1600-2020. These rarer surnames make it possible to follow these families with high fidelity across multiple generations. This database has information for men born 1600-1920 on three social outcomes: occupational status, attainment of higher educational qualifications (0-1)[13], and wealth at death. The FOE records also contain information on the occupational and educational outcomes for the children of marriages. We can use this information to test the assumption made in the opening section: that the matching in marriage was between husband and wife, that wives inherited social capabilities in the same way as did their husbands, and that mothers and fathers contributed equally to child social outcomes such as occupational status or education.

Marital Assortment and Intergenerational Mobility, 1837-2021

Table 3 shows the estimated father-son occupational correlation, and well as that for sons-fathers-in-law and fathers-fathers-in-law, from the parish marriage database 1837-2021 by 40 year periods: 1837-59, 1860-99, 1900-39, 1940-79, and 1980-2021. For the years before 1980 data used is only that where there is a wedding record with occupational status for the groom, father, and father-in-law. But to maximize data numbers for 1980-2021 the correlations are calculated for all son-father, son-father-in-law and father-father-in-law pairs.

[11] https://www.camsis.stir.ac.uk/hiscam/. See Lambert et al., 2013.
[12] https://www.camsis.stir.ac.uk/Data/Britain91.html. See Prandy and Lambert, 2003.
[13] These include attending college, or a military academy, or being a qualified attorney (barrister or solicitor), or a chartered accountant, architect, engineer or surveyor.

Table 3: Occupational Status Correlations, Marriages 1837-2021

Period	Index	ρ_{gf}	se-ρ_{gf}	ρ_{gfinl}	se-ρ_{gfinl}	ρ_{ffinl}	se-ρ_{ffinl}
1837-59	HISCAM	0.631	0.0013	0.480	0.0015	0.439	0.0015
1860-99	HISCAM	0.601	0.0012	0.464	0.0013	0.421	0.0014
1900-39	HISCAM	0.498	0.0021	0.384	0.0022	0.349	0.0023
1940-79	CAMSIS	0.424	0.0039	0.346	0.0041	0.324	0.0044
1980-2021	CAMSIS	0.339	0.0087	0.275	0.0091	0.225	0.0102

Notes: *gf* = groom-father, *gfinl* = groom-father-in-law, *ffinl* = father-father-in-law.

Table 4: Implied values of r and b from table 3

Period	N	r	se – r	b	se – b
1837-1859	343,623	0.771	0.003	0.902	0.004
1860-1899	438,725	0.772	0.003	0.908	0.004
1900-1940	174,474	0.771	0.012	0.909	0.004
1940-1979	47,033	0.816	0.012	0.938	0.017
1980-2021	10,444	0.812	0.034	0.819	0.045

Source: Table 3.

Figure 6: Marital Occupational Status Correlations

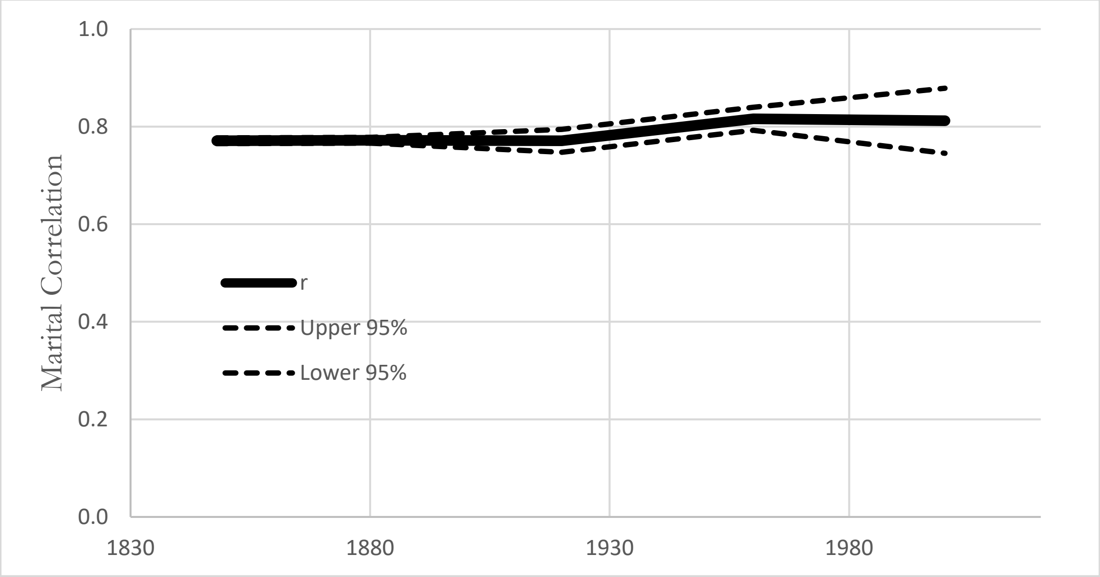

Note: the dashed lines show the 5% confidence interval.

Source: table 4.

Figure 7: Intergenerational Occupational Status Correlations

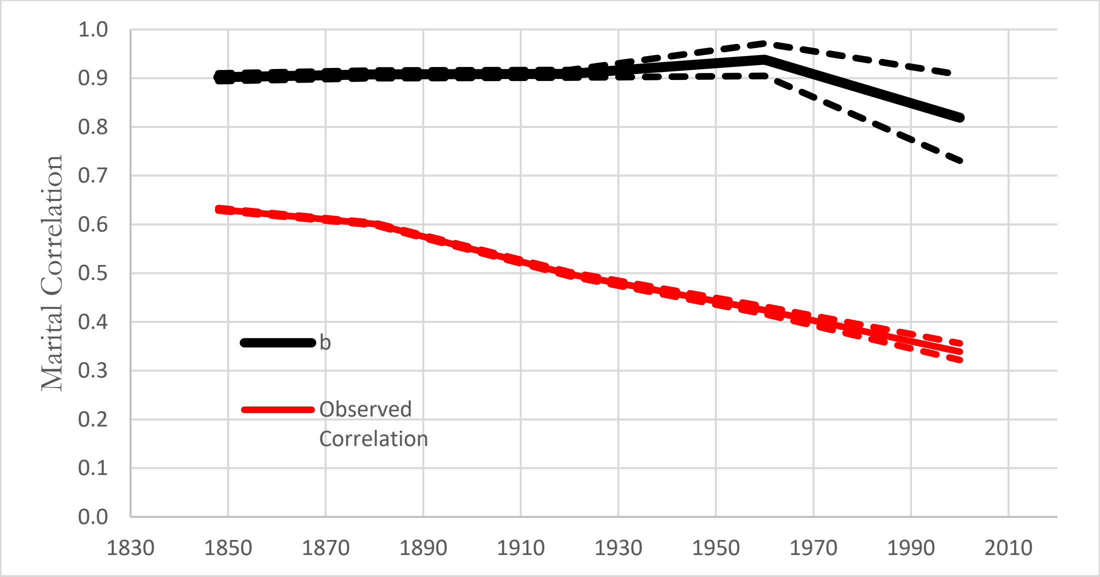

Note: the dashed lines show the 5% confidence intervals.

Source: Tables 3 and 4.

Note that the son-father and son-father-in-law correlations decline substantially and monotonically across these periods. They are strongest for the years 1837-1859 then decline by period to 1980-2021. This might seem the product of greater rates of intergenerational mobility. But it might also be caused just by the changing structure of occupations, and a less clear association of occupational titles with socio-economic status.

From the correlations in table 3 we can estimate underlying marital assortment r, and underlying intergenerational persistence, b, by period. Because these underlying correlations are calculated as the ratio of two normally distributed variables, we bootstrap the standard errors shown. These estimates are shown in table 4. While for 1980-2021 the measured marital correlation in occupational status is 0.329 (standard error 0.008), the table shows a correlation in marriage in terms of underlying occupational status that is close to 0.79 consistently from 1837 to 2021, with small standard errors on the estimate (because of the large amount of data), except for the last period. For the intergenerational correlation b the estimated value is even greater, averaging 0.90. Figures 6 and 7 show the implied marital correlation of occupational status and intergenerational correlation by period, 1837-2021, as well as the 95% confidence bounds.

The parish data on marriages thus suggest strongly an absolute stability in a high degree of status assortment in marriage 1837-2021. The estimated correlation is close to 0.8 throughout. How was it possible men and women could assort in marriage so strongly based on underlying occupational status, when most women had no occupational description in the marriage records before the 1950s? The argument would be that men and women were matching on character traits - intelligence, humor, diligence, honesty – that were themselves highly correlated with social status and with also occupational status. These preferred qualities on both sides of the marriage market led to those of highest social abilities to match similarly and on down the line.

Table 4 estimates underlying marital assortment without any use of the bride's occupational status. As noted we only have this for a non-random subset of brides 1980-2021, which will potentially bias the estimate from the bride's correlation to her father and father-in-law. The raw correlations of bride status to the two fathers are lower than for the grooms: 0.289 and 0.225 versus 0.356 and 0.281. But the estimate of r from the ratio of these correlations is 0.779, with standard error of 0.046. So there is no evidence of any asymmetry here.

Table 5: Implied Marital Correlation using Bride and Groom Literacy, 1837-79

**Relative**	**Bride correlation**	**S.E. correlation**	**Groom correlation**	**S.E. correlation**
Own father	0.1546	0.00007	0.2780	0.00006
Father-in-law	0.1280	0.00007	0.2092	0.00006
Implied marital correlation	0.828	0.0003	0.753	0.0003

Notes: The marital correlation here is the one that obtains if men and women correlate equally in educational status with their fathers.

Table 5 shows for the years 1837-1859 the correlation of bride and groom literacy with father and father-in-law occupational status. If men and women correlate equally in underlying educational status with their fathers, then the estimated educational assortment in marriage is as in the third row of the table. These estimates are not identical, but close. However, we can use (13) to derive an estimate of the marital correlation that is independent of differences in intergenerational correlations in educational status between men and women. The implied marital correlation is 0.790. These estimates, unlike the ones above, uses equally information on literacy among men and women. From (11) or (12) it also follows that the intergenerational correlation b for males is 0.954 of the correlation for women. Thus the assumption of symmetry in inheritance by men and women, if not precisely descriptive, is close to reality.

The further test we can do of the symmetry of men and women in marital assortment and intergenerational transmission of status is to test the relative roles of paternal and maternal grandfathers in predicting child outcomes, using the FOE database. Table 6 shows these estimates for grandchild outcomes of ln wealth at death (both men and women), occupational status (men only), and the higher education indicator (men only). For wealth, there is a clear asymmetry in favor of the paternal grandfather, who has nearly three times the predictive power for grandchild wealth than the maternal grandfather. This is consistent with other evidence for the eighteenth and nineteenth centuries that women inherited significantly less than men of family wealth. But for men's occupational status, or their attainment of higher

Table 6: Symmetry of Mothers versus Fathers in Child Outcomes

Grandfather Status	Ln Wealth	Occupational Rank	Higher Education
**lnWealth**			
Paternal	0.298***		
	(0.029)		
Maternal	0.105***		
	(0.030)		
**Occupational Rank**			
Paternal		0.346***	
		(0.018)	
Maternal		0.312***	
		(0.019)	
**Higher Education**			
Paternal			0.271***
			(0.020)
Maternal			0.289***
			(0.020)
N	577	2,509	2,299
R^2	0.292	0.498	0.252

Note: Source is all the FOE data, for grandchildren born 1650-2000. *** indicates significant at the 1% level.

education, the paternal and maternal grandfathers are equally predictive. There is no statistically significant difference between the predictive effects of maternal versus paternal grandfathers. This implies that the assumption in the marital assortment estimates above that women inherit social capabilities as strongly as men is confirmed. Even in a world where women did not have formal occupations, women played a vital role in the transmission of social status across generations.

Intergenerational Correlations, 1837-2021

The parish data also suggests a powerful persistence across the parent-child generation in underlying social abilities. The persistence here is measured as the ratio of the father to father-in-law correlation to the son to father-in-law correlation.

As table 4 and figure 7 show, based on the parish marriage records shows, the estimated value of the intergenerational correlation is close to 0.9 for the entire interval 1837-1979. The estimated correlation drops to 0.82 in 1980-2021, but because of the smaller amounts of data in this intervals could be as high as 0.91 within the 95% confidence interval.

This is a strong implied persistence. It may, however, be biased upwards in the marriage records by the fact that the occupations of the fathers are given at a similar period in the life course, while the occupations of sons and fathers are given typically 30 years apart in the life course. Over time occupational status correlations between fathers and sons were declining. Perhaps if fathers both formed their occupations in earlier periods they displayed less error attenuation in the measured correlations than do correlations across generations. We can estimate the possible extent of this bias from the rate of decline of observed father correlations over generations. The average upward bias in the estimate of b would be 0.10. So even if this bias operates fully, this would still imply an average value of b of around 0.8.

We should, however, be able to check for biases in the b estimator from (10) by estimating the father-son correlation, but instrumenting for the son status using the father-in-law status. Father in law status as we know correlates well with groom status, but should have no direct effect on the groom's father's status. Table 7 shows these estimates by period. What is apparent in the table is that the average such IV estimate of b is just as high as the ones from (10) above.

The high value of b of 0.9 seems remarkable given standard estimates of intergenerational status correlations. But note that in table 3, for the earliest period 1837-59, the measured correlation using the HISCAM occupational scale is 0.63. In a separate paper, we are able to derive a better HISCAM-like association based occupational scale for the period 1837-1939, using the larger number of marriages we have in the current database than was available to the creators of the HISCAM scale. This new scale increases the father-son

Table 7: Implied values of b from equation (6), and from an IV estimate

Period	N	b	se – b	$\widehat{b_{IV}}$	se – $\widehat{b_{IV}}$
1837-1859	343,623	0.902	0.004	0.920	0.003
1860-1899	438,725	0.908	0.004	0.940	0.003
1900-1940	174,474	0.909	0.004	0.853	0.025
1940-1979	47,033	0.938	0.017	0.986	0.047
1980-2021	10,444	0.819	0.044	0.787	0.038
average		0.895		0.897	

correlation 1837-59 to 0.71. But this 0.71 correlation still incorporates measurement errors. The ranks assigned to occupations have errors, and there is still significant variation in actual occupational status within each occupational descriptor. So we know the true correlation in attained occupational status between fathers and sons 1857-59 has to be significantly greater than 0.71. Thus the plausibility of the 0.90 estimate.

Educational Marital Assortment from the FOE database

The FOE database does not have as much information on social outcomes for the triple of grooms, their fathers, and their father-in-laws as does the marriage database. But we can check where the finding of high status assortment for occupational status generalizes also for educational status using the FOE genealogy data. The measure of educational status is an indicator variable for the attainment of higher education, defined here as enrollment at a university or military academy, attaining a professional qualification such as chartered engineer, chartered accountant, or medical qualification. Most of these educational opportunities were closed to women before the 1880s, and universities such as Oxford and Cambridge did not

formally admit women until the 1920s. So here we are again estimating the underlying educational abilities of brides, not any manifest educational status.

Table 8 shows the estimated father-son occupation correlation, and well as that for sons-fathers-in-law, by 50 year periods, for the FOE database. There is a decline in these correlation over time, but this is an artifact of the database construction where the surnames were chosen to have high or low average social status in 1800, and over time the status of descendants regressed to the mean. This means that the ratio of signal to noise in the early period is greater than in later periods. Using the method here, as can be seen in (9) this will not affect the estimated marital assortment.

Figure 8 shows the implied marital correlation of underlying educational status by 50 year periods, as well as the confidence intervals. Because of much less data in this case these confidence intervals are much wider than with the much larger parish marriages database. But as can be seen this correlation is consistently high, 0.78, all the way from 1750 to 2020. This is consistent with the parish marriage database estimate of marital correlations. The estimate for marriages 1950-2020 is 0.91, but this is not a statistically significant rise from the average of the previous years.

Table 8: Estimated Educational Marital Assortment, FOE, 1750-2020

Period	ρ_{sf}	se-ρ_{sf}	ρ_{sl}	se-ρ_{sl}	r	se-r
1750-99	0.608	0.046	0.494	0.093	0.813	0.165
1800-49	0.527	0.017	0.408	0.028	0.774	0.058
1850-99	0.543	0.009	0.414	0.015	0.763	0.031
1900-49	0.479	0.009	0.314	0.015	0.655	0.034
1950-2020	0.430	0.018	0.392	0.049	0.910	0.119
average					0.783	

Source: FOE database

Figure 8: Implied Marital Correlation on Education, FOE

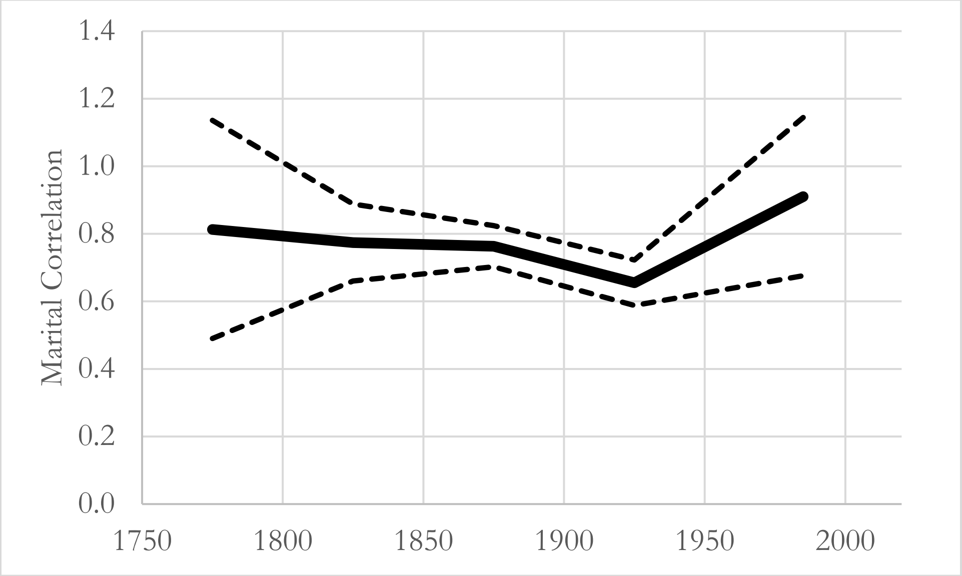

Note: the dashed lines show the 5% confidence intervals.

1754-1859

In the years 1754-1836 grooms and brides were required to sign the parish register, but there was no recording of occupations. We cannot thus measure the underlying marital assortment or intergenerational correlation. We can, however, observe the correlation in literacy between bride and groom. Table 9 shows for three Essex parishes – Aldham, Chelmsford, St Mary, and Colchester, All Saints – the literacy rate of grooms and brides 1754-1839, as well as the estimated correlation in literacy and its standard error. The correlation in literacy changes little 1754-1859.

A complication here is the fact that groom literacy rates were greater than those of brides. A difference in literacy rates between grooms and brides creates a maximum possible correlation in literacy that is less than 1. The maximum possible correlation is given by

$$\rho_{max} = \left(\frac{\theta_f(1-\theta_m)}{\theta_m(1-\theta_f)}\right)^{0.5} \qquad (15)$$

where θ_m, θ_f are male and female literacy rates. As can be seen in table 8 the larger different in male and female literacy rates in earlier years makes the maximum possible correlation lower. The final column of table 8 thus gives the bride-groom correlation as a fraction of its maximum possible value.

As table 9 shows, the adjusted correlation was if anything higher before 1800 than in 1840-59. Assuming the attenuation from the true correlation in educational status by using this 0-1 measure was the same over this time period, the literacy correlation suggest that the strong correlations in underlying occupational and educational status measured 1837-2021 likely extended all the way back to 1754. Marriage in England was likely highly assortative in the years leading up to the Industrial Revolution.

Table 9: Bride-Groom Literacy Correlations, 1754-1859

Place	Period	Number	Groom Lit.	Bride Lit.	Corr. (ρ)	s. e. corr.	Maximum Corr. (ρ_{max})	Adjusted Corr.
Essex	1754-79	1,125	0.600	0.372	0.395	0.028	0.628	0.629
Essex	1780-99	1,079	0.568	0.330	0.374	0.030	0.612	0.612
Essex	1800-19	1,680	0.589	0.379	0.353	0.023	0.653	0.541
Essex	1820-39	1,099	0.702	0.551	0.400	0.025	0.722	0.554
All	1840-59	250,898	0.630	0.499	0.414	0.002	0.765	0.541

Notes: the adjusted correlation is $\frac{\rho}{\rho_{max}}$.

Source: Freereg and Essex Record Office, Church Marriage Registers.

The History of Marriage

We see above that English marriage was highly assortative from at least 1837 onwards, and most likely from even before 1754. We also see that because of the symmetrical influence of mothers and fathers on child outcomes, for outcomes associated with human capital rather than physical capital, that the distribution of these abilities in the upper and lower tails of social abilities would be strongly influenced by marital assortment.

There has been continual speculation on the reasons for the location of the Industrial Revolution in northwest Europe. One recent strain of that speculation associated with, among others, Joel Mokyr, has emphasized the role of "upper tail" human capital in generating technological advance. The Industrial Revolution was created by the high abilities of English engineers and craftsmen, and their interest in, and knowledge of, advances in science. [14]

[14]Mokyr, 2017.

This raises the question of whether one source of greater abilities in the upper tail in England in the Industrial Revolution period was the earlier cultural adoption of highly assortative marriage, associated with the European Marriage Pattern.

The European Marriage Pattern, which present in England from at least 1538 had one important feature for marital assortment. This was that most marriages were between women and men who were legally, and also economically, independent of their parents. Men and women aged 21 or less had to have their father's consent to marry.[15] But the average age of marriage for women was close to 25, and for men 27, so that the great majority of marriages were ones where parental consent was not required. In the sample of marriages used above, 77% had both parties aged 21 and above for marriages 1837-79.

Another sign of the independence of the parties getting married was that throughout these years a significant fraction of women never married, in contrast to other pre-industrial societies where marriage was near universal for women.

By the time of marriage many lower class women had been living independently from their parents as servants in the homes of others. Upper class women were more dependent on their parents, as they typically did not have occupations, and would often receive a financial gift upon marriage from their parents. But these upper class marriages were typically based on an arrangement between a couple, rather than as arrangements between families. Later marriages also allow men and women more time to reveal their realized adult social status than when people marry just after puberty.

In contrast in many other pre-industrial societies marriage for men and women was at much earlier ages, and was much more determined by social conventions and family wishes. The Tsimane, for example, are a shifting-cultivation subsistence agriculture society in Bolivia, has been extensively studied by anthropologists (Godoy et al., 2008, Gurven et al. 2009). The average marriage age was 16 for both men and women, and men and women typically correlate closely in age at marriage. As with other native Amazonians, the strongly preferred marriage partner is a specific cousin, with 75% of couples in the study following this rule. For a man the preferred partner is the daughter of his mothers' brother or his fathers' sister (Godoy, 2008, 204-5). This would imply a limited set of potential marriage partners for each party in a marriage.

[15]In 1987 the age below which parental consent was needed was reduced to 18.

Male suitors ask the parents of a woman for permission to marry the daughter. In a survey asking reasons for marriage among married Tsimane men and women, 38% of women reported the marriage was against their will, while 7% reported they had no other option, and 9% reported it was arranged (Gurven, 2009, 161).[16]

If we assume that in cousin marriage, because of the constraints imposed, a person typically marries a random cousin, what will be the underlying correlation in traits such as education or occupational abilities? The path between cousins is child to parent, sibling to sibling, and parent to child. Empirically the sibling correlation in social characteristics typically equals the parent-child. This implies that the marital correlation in characteristics, r, for cousin marriage will be from (3) above, in social equilibrium

$$r = b^3 = \beta^3 \left(\frac{1+r}{2}\right)^3 \qquad (15)$$

Assuming β, the hereditability of parent traits, has its maximum value of 1, then r = 0.236.[17] Thus cousin marriage, surprisingly, will be much less assortative for social traits than marriage as practiced in England 1754-2021. In line with this among the Tsimane the correlation in years of education at marriage was only 0.14.[18]

Cousin marriage was a surprisingly common marital arrangement in the pre-industrial world. All Muslim communities, for example, seem to have had high shares of marriages taking this form, a traditional that continues to the present. Korotayev argues this stems from the Islamic Law tradition that daughters are entitled to inherit from fathers half of what their brothers inherit (Korotayev, 2000, 400). Cousin marriage keeps such inheritances within the extended family. However, at least in the modern era, cousin marriage is as common among the property-less as among the propertied in Islamic communities. Its association with Islam thus seems more cultural than economic.

However, there is no evidence for the claim of Jack Goody (Goody, 1983) that cousin marriage was common in the Roman Empire in Western Europe. But we do see in the Roman world another common feature of pre-industrial marriage that would reduce marital assortment. This was the practice of parents of contracting marriages for their daughters at

[16] Among men, 11% reported the marriage was against their will, and 14% reported it was arranged
[17] Note that if β=0.7, then with cousin marriage r falls to 0.05.
[18] This was the correlation adjusting for age, since there is a strong age trend. Average years of education for women were 1.24, and for men 2.56 (Godoy, 2008, table 1). The correlation in knowledge of plant names was high, at 0.63, however.

young ages. Keith Hopkins estimates that the average age of marriage in pagan Roman society was well below 18, with even girls as young as 10-11 marrying (Hopkins, 1965).

In marital systems where the match was arranged by the families, suppose there is a correlation, ρ_f, in occupational status between the fathers. In that case the correlation between the spouses will be $\rho_f b^2$, where as above b is the intergenerational correlation and we assume symmetry in the inheritance of occupational abilities between bride and groom. Again from (3) this implies the spousal correlation, r, in social equilibrium is

$$r = \rho_f b^2 = \rho_f \beta^2 \left(\frac{1+r}{2}\right)^2 \quad (16)$$

Assuming again that β=1, and assuming that the fathers match with the correlation of 0.8 we observe in the English marriages 1837-2021, then

$$r = 0.8\left(\frac{1+r}{2}\right)^2 \rightarrow r = 0.4 \quad (17)$$

Thus a marital system where the matching is between families, as represented by the fathers, will again produce a much lower correlation in spouses than observed in England 1837-2021, even if the status matching between fathers is as close as is observed for spouses in England. Matching can only be as strong as is observed for England if the qualities of the spouses themselves that determine the match. But in marital systems where families do the matching, and the spouses are young, these qualities will play little role.

So potentially the development of the European Marriage Pattern in England, where women were mostly living independently of their families by the time of marriage, and were marrying spouses who were contemporaries in age, led to greater inequalities in social abilities and a fatter tail of children with higher talents.[19] It will also be the case that a switch from a regime of little marital assortment across marriage partners to one of high assortment would take many generations to have full effect in terms of the distribution of abilities. We do not know when the European Marriage Pattern was adopted in England. We know it was in place by 1538 when parish records become available in large scale. But knowledge of medieval

[19]Henrich (2020), and Schulz et. al. (2019), argue that the medieval church's prohibitions on cousin marriage weakened kinship structures in Western Europe. This eventually resulted in psychologically different populations when compared to other global regions where cousin marriage preserved intensive kinship ties. But here we are focused just on its effects of marriage systems on the degree of assortment, and hence the distribution of abilities. There is no implication that the European Marriage Pattern changed the general level of abilities.

demographic conditions is too limited to allow an estimate of whether this pattern was an innovation of the 15th century, the 13th century or earlier. But we see above in figure 2 that a a switch to greater assortment even in 1300 would not have full effect in terms of population distributions of abilities until more than 500 years.

Matthew Curtis, who first applied the measures of marital assortment used here to Quebec, 1700-1970, finds a similar degree of marital assortment in the French derived population of Quebec from 1700 on. He also is able to show that women were as important as men for children's educational outcomes (Curtis, 2021). Thus it seems likely that a high degree of marital assortment was a general feature of countries with the European Marriage Pattern across northern Europe.

So there is an intriguing possibility that there is an underlying connection between the European Marriage Pattern, greater assortment in marriage, and the location of the Industrial Revolution in Europe.

Conclusion

We find convincing evidence of a remarkable degree of assortment by underlying occupational and educational status in England all the way from 1754 to 2021, with a likely correlation throughout of 0.8 between marital partners. Notably this correlation is unchanged by the rise in the last 100 years in formal education for women, and in formal employments. Further we show also that women inherited educational and occupational abilities throughout these years as strongly as men, and transmitted these characteristics to children again as strongly as men. This implies that the strong marital assortment substantially reduced social mobility rates, and greatly increased the variance in occupational and educational abilities in the English population. Since pre-industrial marital systems typically involved much less sorting, the development of a new marital pattern that involved greater assortment may play a role in the location and timing of the Industrial Revolution.

BIBLIOGRAPHY

Bruze, Gustaf. 2015. "Male and Female Marriage Returns to Schooling." *International Economic Review*. 56. 10.1111/iere.12100.

Curtis, Matthew. 2021. "The her in inheritance: Matching and mobility in two million Quebec marriages, 1800-1970." Working Paper, Université Libre de Bruxelles.

Gihleb, Rania and Kevin Lang. 2020. "Educational Homogamy and Assortative Mating Have Not Increased." (Research in Labor Economics, forthcoming) NBER Working Paper W229

Godoy, Ricardo, et al. 2008. "Assortative mating and offspring well-being: theory and empirical findings from a native Amazonian society in Bolivia." *Evolution and Human Behavior 29*: 201–210.

Goldin, C. 1992. "The Meaning of College in the Lives of American Women: The Past Hundred Years," Working Paper no. 4099, National Bureau of Economic Research, Cambridge, MA.

Goody, Jack. 1983. *The development of the family and marriage in Europe*. Cambridge: Cambridge University Press.

Greenwood, J, N Guner, G Kocharkov, and C Santos. 2014. "Marry your like: Assortative mating and income inequality." *American Economic Review 104* (5), 348-53.

Gurven, Michael, et. al. 2009. "A Bioeconomic Approach to Marriage and the Sexual Division of Labor." Human Nature, 20:151–183. DOI 10.1007/s12110-009-9062-8.

Haskey, John. 2015. "Marriage rites: trends in marriages by manner of solemnisation and denomination in England and Wales, 1841-2012." In Rebecca Probert, Joanna Miles and Perveez Mody (eds.), *Marriage rites and rights*. London, Hart Publishing.

Henrich, Joseph. 2020. *The WEIRDest People in the World: How the West Became Pyschologically Peculiar and Particularly Prosperous*. New York: Farrar, Straus and Giroux.

Hopkins, M. Keith. 1965. "The Age of Roman Girls at Marriage." *Population Studies*, 18(3): 309-327.

Jepsen, Lisa K, Christopher A Jepsen 2004. "An empirical analysis of the matching patterns of same-sex and opposite-sex couples." *Demography*, 39: 435−453.

Kelly, Morgan, Joel Mokyr, and Cormac O'Grada. 2020. "The Mechanics of the Industrial Revolution." CEPR Discussion Paper No. DP14884.

Korotayev , Andrey. 2000. "Parallel-Cousin (FBD) Marriage, Islamization, and Arabization." *Ethnology*, 39(4): 395-407.

Lambert, Paul S, et al. 2013. "The Construction of HISCAM: A Stratification Scale Based on Social Interactions for Historical Comparative Research," *Historical Methods: A Journal of Quantitative and Interdisciplinary History*, 46:2, 77-89. DOI: 10.1080/01615440.2012.715569

Lefgren, L, and F Mcintyre. 2006. "Examining the Relationship between Women's Education and Marriage Outcomes," *Journal of Labor Economics* 24: 787–830.

Long, Jason, and Joe Ferrie. 2013. "Intergenerational occupational mobility in Great Britain and the United States since 1850." *American Economic Review*, *103*(4), 1109-37.

Mare, R. D. 1991. "Five Decades of Educational Assortative Mating," *American Sociological Review*, 56(1), 15–32.

Mokyr, Joel. 2018. "Bottom-up or top-down? The origins of the Industrial Revolution." *Journal of Institutional Economics*, 14: 1003-24.

Perez, Santiago. 2019. "Intergenerational Occupational Mobility Across Three Continents" The Journal of Economic History*, 79(2): 383-416.*

Prandy, K, and Paul S Lambert. 2003. "Marriage, Social Distance and the Social Space: An alternative derivation and validation of the Cambridge Scale." *Sociology*, 37(3): 397-411.

Qin, Zhaoxiong. 2001. 'Rethinking Cousin Marriage in Rural China." *Ethnology, 40(4)*: 347-360.

Schulz, Jonathan F., Duman Bahrami-Rad, Jonathan P. Beauchamp, and Joseph Henrich. 2019. "The Church, intensive kinship, and global psychological variation." *Science* 366, no. 6466: eaau5141.

Schwartz, Christine R. 2013. Trends and Variation in Assortative Mating: Causes and Consequences. *Annual Review of Sociology, 39(1)*: 451-470.

Schwartz, C. and R. Mare. 2005. Trends in educational assortative marriage from 1940 to 2003. *Demography 42*(4): 621-646.

Shaw, Brent D. and Richard P. Saller. 1984. "Close-Kin Marriage in Roman Society?" *Man*, 19(3), 432-444.

Squicciarini, M P, and N Voigtländer. 2015. "Human Capital and Industrialization: Evidence from the Age of Enlightenment", *Quarterly Journal of Economics* 130(4), 1825–83.

Young, Michael 1958. *The Rise of the Meritocracy 1870-2033: An Essay on Education and Equality*. London: Thames & Hudson.

Xi, Song, Catherine G. Massey, Karen A. Rolf, Joseph P. Ferrie, Jonathan L. Rothbaum, Yu Xie. 2020. "Long-term decline in intergenerational mobility in the United States since the 1850s." *Proceedings of the National Academy of Sciences*, 117(1): 251 258; DOI: 10.1073/pnas.1905094116.
